# Neuron‐targeted 2‐deoxyglucose‐dendrimer‐rosiglitazone nanotherapy mitigates neuroinflammation and cognitive deficits in pediatric traumatic brain injury

**DOI:** 10.1002/btm2.70053

**Published:** 2025-07-22

**Authors:** Aqib Iqbal Dar, Zhi Zhang, Shamila Gopalakrishnan, Rishi Sharma, Anunay James Pulukuri, Anu Rani, Anubhav Dhull, Joan Castaneda Gonzalez, Tia Atoui, Yara Mashal, Zahrah Naseer, Julia Calmi, Anjali Sharma

**Affiliations:** ^1^ Department of Chemistry, College of Arts and Sciences Washington State University Pullman Washington USA; ^2^ Department of Natural Sciences, College of Arts, Sciences, and Letters University of Michigan–Dearborn Dearborn Michigan USA

**Keywords:** glyco‐dendrimer, neuroinflammation, neuron targeting, rosiglitazone, targeted drug delivery, traumatic brain injury

## Abstract

Traumatic brain injury (TBI) remains a major global health challenge, characterized by high morbidity and mortality rates. Despite advances in neuroscience, the blood–brain barrier (BBB) limits the effectiveness of potential neuroprotective treatments. Recent nanotechnology breakthroughs have led to smart drug delivery systems that can cross the BBB and target injured brain areas. However, achieving the specificity needed to deliver therapies to affected neurons remains a challenge. In previous work, we developed a mixed‐layered dendrimer functionalized with 2‐deoxyglucose (2DG‐D) for selective neuronal drug delivery. In this study, we explore the therapeutic potential of rosiglitazone (Rosi) for pediatric TBI by creating a 2DG‐D‐Rosi nanosystem, where Rosi is conjugated to 2DG‐D to improve its solubility, bioavailability, and targeted delivery to injured neurons. In vitro, 2DG‐D‐Rosi demonstrated high neuronal uptake, sustained drug release, and excellent biocompatibility. It significantly reduced neuronal apoptosis, reactive oxygen species formation, pro‐inflammatory cytokine expression, and caspase activity, outperforming free Rosi. In vivo, using a pediatric TBI mouse model, 2DG‐D‐Rosi improved neuronal targeting, reduced neuroinflammation, and enhanced behavioral outcomes. This research highlights 2DG‐D‐Rosi as a promising nanotherapeutic platform for precise TBI treatment and sets the stage for developing more effective therapies for this challenging condition.

**Translational Impact Statement**

This study presents a promising nanotherapeutic strategy for traumatic brain injury (TBI) using 2‐deoxyglucose‐rosiglitazone (2DG‐D‐Rosi), a dendrimer‐based targeted drug delivery system. By improving rosiglitazone's solubility, bioavailability, and neuronal specificity, 2DG‐D‐Rosi effectively accumulates at brain injury sites within the neurons from systemic administration. In vitro, it reduces neuronal apoptosis, oxidative stress, and inflammation, while in vivo, it enhances neuroprotection and functional recovery in a pediatric TBI mouse model. This work advances precision nanomedicine for TBI and lays the foundation for broader applications in neurodegenerative disease treatment.

## 1 INTRODUCTION

Traumatic brain injury (TBI) is a life‐threatening global neurological condition with no effective treatment currently available to mitigate its long‐term consequences. Alarmingly, nearly half of the global population is expected to experience a TBI at some point in their lifetime.^1^ It is estimated that there are 27–69 million cases of TBI annually worldwide, with approximately 69,000 related deaths in the United States alone in 2021, averaging 190 deaths per day.^2^, ^3^ The Lancet Neurology has reported an estimated global economic loss of $400 billion resulting from TBI, imposing a significant financial burden on society, particularly low‐ and middle‐income families.^4^ TBI typically results from a primary injury due to physical trauma to the brain, which sets off a cascade of damaging biological pathways, including neuroinflammation, oxidative stress, autophagy, and neuronal loss.^5^, ^6^ These processes exacerbate neuronal and glial cell damage, primarily through the activation of microglia and astrocytes, which overexpress neuroinflammatory mediators.^7^, ^8^

Infants or toddlers who suffer from TBI are at high risk of developing neurodegenerative and psychiatric diseases later in life.^9^ Although the mortality rate of pediatric TBI has declined with advances in medical care, more children and adolescents are living with TBI‐related cognitive and emotional impairments. There is an unmet need to develop effective treatments for pediatric patients.

Advancements in nanotechnology and biomedical engineering, however, have introduced new opportunities for developing sophisticated nano‐delivery systems that can target diseased sites in the brain.^10^, ^11^, ^12^ These systems offer the potential to deliver therapeutic agents directly to the key cells involved in injury following TBI. One of the most significant challenges, however, lies in achieving selective targeting of neurons at the site of brain injury while sparing healthy ones. This precision is essential to deliver drugs specifically to disease‐associated neurons, rescuing them and mitigating further injury. Achieving this could revolutionize TBI treatment strategies. We have recently reported a mixed‐layered dendrimer functionalized with 2‐deoxyglucose (2DG‐D) at its periphery, designed to selectively target and deliver drugs to neurons at brain injury sites following systemic administration.^13^ Constructed primarily from Generally Recognized As Safe (GRAS) and biocompatible materials, this 2DG‐D dendrimer leverages a highly efficient, reproducible, and robust synthetic process, facilitating smooth translation to advanced stages of development. Systemically administered 2DG‐D targets neurons at the site of brain injury without accumulating in healthy brain regions and off‐target organs in a mouse model of pediatric TBI, making it a versatile platform for neuronal drug delivery for brain diseases.^13^

Rosiglitazone (Rosi) is an anti‐diabetic drug from the thiazolidinedione class, which functions as an insulin sensitizer by binding to peroxisome proliferator‐activated receptor (PPAR).^14^ Rosi has been shown to regulate astrocyte polarization and neuroinflammation by increasing the expression of peroxisome proliferator‐activated receptor gamma (PPARγ), providing neuroprotection against TBI.^15^ Furthermore, Rosi is shown to provide neuroprotection via suppression of autophagy and apoptosis and anti‐oxidant action in the cortical neurons.^16^ Initially, Rosi was primarily utilized as a standalone drug in managing type 2 diabetes.^17^ However, despite its effectiveness, the drug was banned following a meta‐analysis in 2007 that demonstrated its association with increased heart attack and death.^18^ Rosi also exhibits poor aqueous solubility with low bioavailability and other off‐target effects, which limit its clinical use and effectiveness in TBI.^19^, ^20^

In this study, we hypothesized that Rosi conjugated to 2DG‐D (2DG‐D‐Rosi) could selectively target neurons at the site of brain injury, enhancing its neuronal bioavailability and therapeutic efficacy while mitigating early pathological events following TBI. We achieved the following: (1) successfully synthesized and conducted a comprehensive characterization of the 2DG‐D‐Rosi conjugate; (2) investigated its in vitro neuronal uptake mechanisms and therapeutic potential; and (3) demonstrated in vivo neuron‐targeting ability of systemically administered 2DG‐D‐Rosi and its efficacy in ameliorating neuroinflammation and neuronal loss and improving behavioral outcomes in juvenile mice subjected to TBI.

## 2 EXPERIMENTAL SECTION

### 2.1 Materials

All starting materials and reagents (Rosi, formaldehyde, triethylamine [TEA], 1‐[3‐(dimethylamino)propyl]‐3‐ethylcarbodiimide [EDC], 4‐(dimethylamino)pyridine [DMAP], *N*,*N*‐diisopropylethylamine [DIPEA], copper sulfate pentahydrate (CuSO_4_ • 5H_2_O), and sodium ascorbate [NaAs]) were sourced from Sigma‐Aldrich US, Merck, or Thermo Fisher Scientific and were used as received. Solvents were of analytical grade and used as received without additional purification. Thin‐layer chromatography (TLC) was performed using silica‐coated aluminum sheets with F_254_ fluorescent indicator (purchased from Merck). Column chromatography was performed using silica gel 60 (70–230 mesh) as the stationary phase. Spectra/Por dialysis membranes from Repligen were used for the dialysis. Phosphate‐buffered saline (PBS), poly‐L‐lysine, 4′,6‐diamidino‐2‐phenylindole (DAPI), 3‐(4,5‐ dimethylthiazol‐2‐yl)‐2,5 diphenyltetrazolium bromide (MTT), and Triton X‐100 were procured from Aaron Chemicals. CATH.a, Eosinophilic cationic protein clone EOC 20 (EOC 20) cell line was obtained from American Type Culture Collection (ATCC), United States. Dulbecco's Modified Eagle's Medium (DMEM) and Roswell Park Memorial Institute (RPMI‐1640) were purchased from Gibco; Fetal Bovine Serum (FBS) were obtained from Gibco Scientific, and VectaShield antifade mounting media was obtained from VectorLabs. Rat red blood cells (RBCs) were purchased from Innovative Research. Tumor Necrosis Factor‐alpha (TNF‐α)‐(Interleukin 6) Il‐6 enzyme‐linked immunosorbent assay (ELISA) kits were obtained from BioLegend, USA. Caspase activity stain was procured from Revvity. Fluorescein Isothiocyanate (FITC) ApoScreen® Annexin V Apoptosis Kit was procured from Southern Biotech. All these above‐mentioned reagents were used as such.

### 2.2 Instrumentation/methods

Nuclear Magnetic Resonance (NMR) spectra were recorded using a Bruker 500 MHz spectrophotometer. The synthesized compounds were dissolved in appropriate deuterated solvents such as chloroform‐d (CDCl3‐d), dimethyl sulfoxide‐d6 (DMSO‐d6), deuterium oxide (D2O) to obtain spectral data for proton NMR (^1^H NMR, 500 MHz) and carbon NMR (^13^C NMR, 125 MHz) at 25°C. Chemical shifts (*δ*) for ^1^H‐NMR were reported in parts per million (ppm) using the solvent peak as a reference. The coupling constants (*J*) were expressed in hertz (Hz). The following abbreviations are used to describe the signal patterns: s (singlet), d (doublet), t (triplet), q (quartet), and m (multiplet). The molecular weights of the small molecules were determined using a Bruker micrOTOF II spectrometer with electrospray ionization (ESI) as the ionization source. For dendrimers and dendrimer conjugates, mass analysis was performed via direct infusion using ESI on a Bruker MALDI‐TOF instrument employing trans‐2‐[3‐(4‐*tert*‐butylphenyl)‐2‐methyl‐2‐propenylidene]malononitrile as the matrix.

High‐performance liquid chromatography (HPLC) was performed using a Waters Acquity Arc HPLC system equipped with binary pumps, a 2998 photodiode array detector, and a 2475 fluorescence detector. Data was analyzed using the Waters Empower software. Chromatographic separation was achieved with a Waters Symmetry C_18_ column (5 μm, 4.6 × 250 mm) using the gradient flow method at a flow rate of 1 mL/min. The mobile phase consisted of water containing 0.1% trifluoroacetic acid (Solvent A) and acetonitrile containing 0.1% trifluoroacetic acid (Solvent B). The gradient was initiated at 90:10 (A:B), adjusted to 40:60 (A:B) over 20 min, maintained at 40:60 (A:B) for 5 min, and returned to the initial ratio of 90:10 (A:B) by 45 min. The cyanine 5 (Cy5) labeled 2DG‐D‐Rosi was analyzed using a different gradient method. The gradient was initiated at 90:10 (A:B), adjusted to 50:50 (A:B) for over 20 min, then changed to 10:90 (A:B), and maintained for 18 min, before returning to the initial ratio of 90:10 (A:B) by 40 min. The Rosi, Rosi derivatives, 2DG‐D, 2DG‐D‐Rosi, and their derivatives were detected at 210, 233, and 254 nm wavelengths, while the Cy5 labeled dendrimer conjugate was specifically monitored at 650 nm. The HPLC column temperature was maintained at 25°C to ensure consistent retention times and optimal peak resolutions.

The size distribution and zeta potential of the dendrimer and dendrimer‐drug derivatives were analyzed using dynamic light scattering (DLS) on a Malvern Zetasizer Nano 90 (Westborough, MA, USA). For size distribution analysis, the samples were prepared at a concentration of 0.1 mg/mL in Milli‐Q water. For zeta potential measurements, the samples were dissolved at a concentration of 0.2 mg/mL in 10 mM sodium chloride solution. All measurements were performed in triplicate to ensure accuracy.

### 2.3 Synthesis protocols

Compound **5** (2DG‐D) and Compound **9** (2DG‐D‐hexyne with 10 alkyne arms) were synthesized using our previously published protocols.^13^

#### 2.3.1 Synthesis of Compound **2**

Rosi **1** (250 mg, 0.516 mmol) was dissolved in anhydrous *N*,*N*‐dimethylformamide (DMF, 3 mL) and treated with formaldehyde (33.6 mg, 11.2 mmol) and TEA (1.6 mL 11.2 mmol). The reaction mixture was stirred in an inert environment for 10 h, and the completion of the reaction was confirmed by TLC. The mixture was then diluted with DCM (300 mL), and the organic layer was sequentially washed with water (20 mL × 2) and brine (20 mL × 2), dried with anhydrous Na_2_SO_4_, filtered, and evaporated in vacuo. The crude product was purified using silica gel flash column chromatography (ethyl acetate/hexane, 40:60 [v/v]) to afford Compound **2** in 87% yield (off‐white powder).

^*1*^ *H NMR* (500 MHz, DMSO‐d_6_) *δ* 8.12–8.04 (m, 1H), 7.50 (t, *J* = 8.1 Hz, 1H), 7.10–7.03 (m, 2H), 6.88–6.79 (m, 2H), 6.65 (d, *J* = 8.6 Hz, 1H), 6.60–6.52 (m, 1H), 5.65 (t, *J* = 5.6 Hz, 1H), 4.71 (d, *J* = 7.4 Hz, 1H), 4.09 (q, *J* = 6.0 Hz, 2H), 3.99–3.82 (m, 3H), 3.67–3.55 (m, 1H), 3.19–3.02 (m, 4H), 2.98 (d, *J* = 13.9 Hz, 1H).

^*13*^ *C NMR* (125 MHz, DMSO‐d_6_) *δ* 176.1, 171.2, 158.5, 158.0, 148.0, 137.8, 131.9, 131.9, 127.5, 127.2, 114.5, 114.4, 112.0, 106.2, 70.1, 68.2, 66.3, 66.1, 65.7, 64.0, 48.9, 38.8, 38.6, 37.5.

*ESI‐MS*: *m*/*z*: calculated for C_19_H_21_N_3_O_4_S: 387.13; found as [M + H]^+^: 388.13.

#### 2.3.2 Synthesis of Compound **4**

In a round bottom flask, 6‐azido‐hexanoic acid (200 mg, 0.516 mmol) was dissolved in anhydrous DMF (5 mL) and mixed with 1‐ethyl‐3‐(3‐dimethylaminopropyl) carbodiimide (160.21 mg, 1.032 mmol) under argon atmosphere. After stirring for 3 min, Compound 2 (162 mg, 1.032 mmol) and 4‐dimethylaminopyridine (DMAP, 56.7 mg, 0.46 mmol) were added to the reaction mixture. On completion, the reaction mixture was diluted with DCM (300 mL) and the organic layer was washed with water (20 mL × 2) and brine. The organic layer was then dried using anhydrous Na_2_SO_4_, filtered, and evaporated under vacuum. The crude product was purified using silica flash column chromatography [ethyl acetate/hexane, 20:80 (v/v)] to afford Compound 4 as an off‐white oil in 44.7% yield.

^*1*^ *H NMR* (500 MHz, DMSO‐d_6_) *δ* 8.07 (dd, *J* = 4.9, 1.9 Hz, 1H), 7.57–7.44 (m, 1H), 7.10 (d, *J* = 8.5 Hz, 2H), 6.95–6.82 (m, 2H), 6.63 (d, *J* = 8.6 Hz, 1H), 6.56 (dd, *J* = 7.0, 4.9 Hz, 1H), 4.52 (d, *J* = 11.4 Hz, 1H), 4.36 (d, *J* = 11.4 Hz, 1H), 4.10 (t, *J* = 5.9 Hz, 2H), 3.88 (t, *J* = 5.9 Hz, 2H), 3.32–3.25 (m, 3H), 3.19 (d, *J* = 13.8 Hz, 1H), 3.10 (d, *J* = 13.9 Hz, 1H), 3.06 (s, 3H), 2.32 (t, *J* = 7.2 Hz, 2H), 1.63–1.43 (m, 4H), 1.38–1.21 (m, 2H).

^*13*^ *C NMR* (125 MHz, DMSO‐d_6_) *δ* 172.4, 158.5, 158.2, 148.0, 137.8, 132.1, 126.6, 114.5, 112.0, 106.2, 66.4, 65.7, 50.9, 48.9, 38.8, 37.5, 33.7, 28.3, 26.0, 24.4.

*ESI‐MS*: *m*/*z*: calculated for C_24_H_28_N_6_O_5_S: 512.18; found as [M + H]^+^: 513.19.

#### 2.3.3 Synthesis of 2DG‐D‐hexyne (Compound **7**)

5‐Hexynoic acid **6** (11.5 mg, 14.0 eq, 0.098 mmol) was dissolved in dry DMF (6 mL). The coupling reagents EDC • HCl (8.35 mg, 0.078 mmol) and DMAP (6.0 mg, 0.009 mmol) were added, and stirring was continued at room temperature (RT) for 20 min. To this reaction vessel, 2DG‐D dendrimer **5** (150 mg, 1.0 eq, 0.007 mmol) in 3 mL of dry DMF was added dropwise for 20 min and allowed to stir for 24 h. The progress of the reaction was monitored using HPLC. Upon completion, the reaction mixture was purified by dialysis using a 1 kDa dialysis membrane first in DMF for 16 h, followed by dialysis in deionized (DI) water for an additional 16 h. The product was then lyophilized to obtain Compound **7** in 84% yield.

^*1*^ *H NMR* (500 MHz, DMSO‐d_6_) *δ* 8.52 (s, 8H), 8.31–7.73 (m, 53H), 7.24 (s, 16H), 5.26–5.08 (m, 9H), 5.00–4.76 (m, 61H), 4.69–4.33 (m, 148H), 4.28–4.03 (m, 64H), 3.91–3.79 (m, 108H), 3.73–3.63 (m, 138H), 3.61–3.50 (m, 885H), 3.23–2.98 (m, 67H), 2.90–2.60 (m, 44H), 2.46 (t, *J* = 7.2 Hz, 28H), 2.35–2.08 (m, 56H), 1.98–1.71 (m, 57H), 1.56–1.40 (m, 24H).

^*13*^ *C NMR* (125 MHz, DMSO‐d_6_) *δ* 173.2, 166.0, 152.2, 144.3, 140.4, 129.7, 124.7, 106.7, 97.2, 97.1, 73.6, 72.3, 72.1, 72.1, 71.1, 70.5, 70.4, 70.3, 70.3, 70.2, 70.2, 70.1, 70.1, 70.0, 70.0, 69.5, 69.4, 69.2, 69.2, 68.8, 68.4, 68.2, 66.2, 66.0, 64.1, 64.0, 61.5, 50.9, 49.8, 38.3, 38.2, 33.8, 28.4, 26.1, 26.0, 25.6, 24.5.

#### 2.3.4 Synthesis of 2DG‐D‐Rosi (Compound **8**)

A solution of 2DG‐D‐hexyne **7** (50 mg, 1.0 eq, 0.0021 mmol) in DMF (0.2 mL) was added to a stirred solution of Compound **4** (0.015 mg, 14 eq, 0.030 mmol) in DMF (0.1 mL) in a reaction vial. This was followed by a sequential addition of CuBr (10 mol % per acetylene, 12 mg), *N*,*N*,*N*′,*N*′′,*N*′′‐Pentamethyldiethylenetriamine (PMDETA, 4.5 mg), and Tris(3‐hydroxypropyltriazolylmethyl)amine (THPTA 1.5 mg, catalytic). The reaction mixture was then stirred at RT for 10 h. Upon completion, purification was performed using a 1 kDa dialysis membrane against DI water for 12 h. The aqueous solution was then lyophilized to obtain Compound **8** in 95% yield (brown solid).

^*1*^ *H NMR* (500 MHz, DMSO‐d_6_) *δ* 8.47 (t, *J* = 5.6 Hz, 8H), 8.13–7.98 (m, 52H), 7.86–7.79 (m, 29H), 7.48 (t, *J* = 7.8 Hz, 15H), 7.18 (s, 16H), 7.09 (d, *J* = 8.2 Hz, 29H), 6.85 (d, *J* = 8.2 Hz, 28H), 6.62 (d, *J* = 8.6 Hz, 14H), 6.54 (t, *J* = 6.0 Hz, 14H), 4.88–4.77 (m, 32H), 4.59–4.36 (m, 180H), 4.35–4.17 (m, 90H), 4.16–3.99 (m, 141H), 3.97–3.53 (m, 685H), 3.40–3.23 (m, 191H), 3.16–2.96 (m, 178H), 2.83–2.53 (m, 119H), 2.39–2.04 (m, 146H), 1.91–1.66 (m, 115H), 1.55–1.35 (m, 64H), 1.30–1.14 (m, 47H).

^*13*^ *C NMR* (125 MHz, DMSO‐d_6_) *δ* 173.1, 172.4, 158.5, 158.1, 152.2, 148.0, 144.2, 137.8, 132.0, 124.7, 122.2, 114.4, 112.0, 106.7, 106.2, 97.2, 97.1, 73.5, 72.1, 70.4, 70.3, 70.2, 70.2, 70.2, 70.1, 70.1, 70.0, 69.4, 69.2, 69.2, 68.8, 68.4, 68.2, 66.6, 66.2, 66.0, 65.7, 63.9, 61.5, 49.7, 49.4, 48.9, 40.4, 40.4, 40.3, 40.2, 40.1, 40.0, 39.9, 39.8, 39.6, 39.4, 38.3, 37.5, 33.6, 33.4, 29.8, 25.7, 25.6, 25.1, 24.8, 24.2.

*MALDI‐TOF*: *m*/*z*: calculated for Compound **8**: 30,546; found as: 30,486.

#### 2.3.5 Synthesis of 2DG‐D‐Cy5 (Compound **10**)

Compound **9** (50 mg, 1.0 eq, 0.003 mmol) was dissolved in DI water (1 mL) in a reaction vial and stirred at RT. Cy5 Azide (7.7 mg, 2.5 eq., 0.0075 mmol) dissolved in 1 mL of DMF was added to the stirred solution. The click reaction was initiated by adding a solution of CuSO_4_ • 5H_2_O (10 mol% per acetylene, dissolved in 0.1 mL DI water), followed by the addition of NaAs (15 mol% per acetylene, dissolved in 0.1 mL DI water) over 2 min. The reaction mixture was then stirred at 40°C for 15 h. The progress of the reaction was monitored using HPLC. Upon completion, purification was performed by dialysis using a 1 kDa dialysis membrane against DI water for 15 h. The purified product was lyophilized to obtain Compound **10** in 90% yield.

^*1*^ *H NMR* (500 MHz, DMSO‐d_6_) *δ* 8.47 (s, 8H), 8.36 (t, *J* = 13.0 Hz, 5H), 8.19–7.75 (m, 56H), 7.65 (s, 7H), 7.39–7.27 (m, 6H), 7.18 (s, 16H), 7.02 (s, 2H), 6.65–6.52 (m, 7H), 6.30 (d, *J* = 13.3 Hz, 7H), 5.31–5.07 (m, 16H), 5.04–4.63 (m, 70H), 4.61–4.20 (m, 154H), 4.20–3.95 (m, 86H), 3.95–3.41 (m, 1042H), 3.29–2.94 (m, 71H), 2.80 (s, 6H), 2.62 (s, 17H), 2.42–2.32 (m, 19H), 2.27–2.00 (m, 39H), 1.92–1.77 (m, 39H), 1.68 (s, 43H), 1.62–1.11 (m, 71H).

#### 2.3.6 Synthesis of 2DG‐D‐Rosi‐Cy5 (Compound **11**)

A solution of 2DG‐D‐Cy5 **10** (30 mg, 1.0 eq, 0.0011 mmol) dissolved in DMF (0.2 mL) was added to a stirred solution of Compound **4** (4.7 mg, 10 eq, 0.030 mmol) in DMF (0.1 mL) in a reaction vial. This was followed by the sequential addition of CuBr (10 mol% per acetylene, 12 mg), PMDETA (4.5 mg), and THPTA (1.5 mg, cat.). The reaction mixture was then stirred at RT for 10 h. Upon completion, the mixture was purified by dialysis against DMF and DI water for 12 h using a 1 kDa dialysis membrane. The aqueous solution was lyophilized to afford Compound **11** in 95% yield (blue cotton‐like solid).

^*1*^ *H NMR* (500 MHz, DMSO‐d_6_) *δ* 8.47 (s, 8H), 8.36 (t, *J* = 13.0 Hz, 10H), 8.10–8.00 (m, 37H), 7.98–7.75 (m, 55H), 7.64 (t, *J* = 6.8 Hz, 10H), 7.47 (s, 14H), 7.32 (t, *J* = 6.4 Hz, 11H), 7.18 (s, 16H), 7.09 (s, 15H), 6.80 (s, 14H), 6.66–6.46 (m, 28H), 6.30 (d, *J* = 13.5 Hz, 8H), 5.30–4.69 (m, 83H), 4.62–4.37 (m, 143H), 4.35–4.19 (m, 44H), 4.17–3.95 (m, 111H), 3.93–3.70 (m, 156H), 3.71–3.33 (m, 669H), 3.49 (s, polyethylene glycol (PEG), 3.19–2.85 (m, 155H), 2.71–2.51 (m, 80H), 2.33 (dd, *J* = 16.6, 8.7 Hz, 62H), 2.26–2.07 (m, 62H), 2.03 (t, *J* = 7.3 Hz, 33H), 1.91–1.64 (m, 146H), 1.63–1.40 (m, 87H), 1.38–1.15 (m, 78H), 1.07 (d, *J* = 6.6 Hz, 55H).

^*13*^ *C NMR* (125 MHz, DMSO‐d_6_) *δ* 173.5, 173.1, 172.4, 166.0, 158.5, 154.7, 152.2, 148.1, 144.2, 140.3, 137.8, 131.9, 129.7, 124.7, 122.7, 114.2, 112.0, 110.6, 106.7, 106.3, 97.2, 97.1, 73.6, 72.1, 70.4, 70.3, 70.3, 70.2, 70.1, 70.1, 70.0, 69.4, 69.2, 69.2, 68.8, 68.4, 68.2, 66.3, 66.1, 64.0, 61.5, 49.8, 49.4, 49.0, 48.9, 38.8, 38.3, 36.2, 35.6, 35.3, 33.5, 28.9, 27.6, 27.4, 26.1, 25.6, 25.3, 25.1, 24.8, 12.6.

### 2.4 In vitro drug release and stability studies

#### 2.4.1 In vitro drug release studies

The studies to evaluate the release of Rosi from the 2DG‐D‐Rosi dendrimer were carried out under plasma conditions (pH 7.4, PBS and 2% FBS in PBS) and intracellular conditions (pH 5.5, citrate buffer with esterase from porcine liver). Accurately, 2 mg of 2DG‐D‐Rosi was dissolved in 1 mL of the above buffer solutions separately and incubated at 37°C while shaking the solution mixtures continuously to simulate the natural physiological state. The sample solution of 100 μL was taken out at regular intervals and transferred to Eppendorf, quenched immediately with 100 μL of methanol, and stored at −20°C until analyzed by HPLC. Rosi release was measured with respect to the calibration curve of free Rosi via HPLC.

#### 2.4.2 2DG‐D‐Rosi formulation stability studies

The formulation of 2DG‐D‐Rosi was developed in PBS at the concentration of 50 mg/mL, which was filtered through 0.4 μm sterile filters. The formulations were stored at 25°C (RT) and 4°C. The stability and shelf life of 2DG‐D‐Rosi were analyzed using HPLC at predetermined time points (0 h, 1 day, 7 days, 14 days, and 28 days).

### 2.5 In vitro studies

#### 2.5.1 Hemocompatibility and cytocompatibility of 2DG‐D‐Rosi

For the intended in vitro and in vivo use of the developed 2DG‐D‐Rosi dendrimer, ex vivo hemocompatibility and in vitro cellular compatibility studies were carried out. For evaluating the effect of 2DG‐D‐Rosi on the RBCs, a hemolysis assay was carried out. In brief, a 1:3 (RBC:1× PBS) solution was taken in the microcentrifuge tubes (MCTs) and kept at 37°C for 1 h. Following this, the same volumes of 2DG‐D (1000 μg/mL), 2DG‐D‐Rosi, or Rosi (100, 500, and 1000 μg/mL) were added to the respective MCTs and incubated for ~2 h at 37°C in an incubator shaker at ~100 rpm. After the incubation, the samples were centrifuged at 5000 × g for 10 min; following this, 200 μL supernatant was taken and absorbance was recorded at 540 nm using a microplate reader (Synergy H1 hybrid multi‐mode microplate reader, BioTek Instruments). RBCs treated with PBS and Triton‐X‐100 were kept as a negative control and positive control, respectively. The percentage hemolysis was calculated using equation 1. All the measurements were done in triplicates, and the hemolytic index was determined from the recommendation by ASTM E2524‐08 standard.^21^, ^22^

(1) $$\text{Hemolysis}\;\left(\%\right)=\frac{\text{absorbance of test sample}}{\text{absorbance of positive control}}\times 100.$$

Cytocompatibility studies were carried out using the MTT assay as described in our previous work.^13^ For this, CATH.a, EOC 20, and HUVEC cells seeded in a 96‐well plate at a density of 1 × 10^4^ cells/well were incubated with different concentrations of 2DG‐D (1000 μg/mL), 2DG‐D‐Rosi, or Rosi (100, 500, and 1000 μg/mL) for 24 h in a CO_2_ incubator. After this, all the treated cells were incubated with MTT for 3 h until the purple‐colored formazan crystals appeared. For dissolving these crystals, cell culture grade DMSO was added to each of the wells and incubated for 15 min. Finally, the absorbance was recorded at 570 nm using Synergy H1 hybrid multi‐mode microplate. The percentage cytocompatibility was calculated using Equation (2). All the measurements were done in triplicates with proper controls.

(2) $$\text{Cell viability}\;\left(\%\right)=\frac{\text{absorbance of sample}}{\text{absorbance of control}}\times 100.$$

#### 2.5.2 In vitro neuronal uptake of 2DG‐D‐Rosi

Prior to utilizing 2DG‐D‐Rosi dendrimers in an in vivo TBI mouse model, we investigated their cellular uptake and internalization under in vitro conditions to elucidate the underlying mechanism of uptake in CATH.a neuronal cells. These studies were performed with minor modifications following our previously published protocols.^13^ Briefly, CATH.a cells were cultured and seeded at a density of 2 × 10^5^ cells/well onto poly‐L‐lysine‐coated glass cover slips. To assess the impact of specific cellular trafficking pathways, the cells were treated with various inhibitors: methyl‐β‐cyclodextrin (MβCD, ~2 mM), chlorpromazine (CPZ, 30 μM), cytochalasin B (20 μM), and phloretin (~1 mM).^13^ Treatments were conducted for 2 h at 37°C in a CO_2_ incubator. Post‐treatment, the inhibitor‐containing medium was aspirated, and the cells were washed with 1× PBS before being incubated with 2DG‐D‐Rosi‐Cy5 for 6 h under identical incubation conditions. Following incubation, the cells were washed with cold PBS and fixed with 4% paraformaldehyde (PFA), prepared by mixing equal volumes of 8% PFA and 2× 60 mm 1,4‐piperazinediethanesulfonic acid (PIPES), 25 mm 4‐(2‐hydroxyethyl)piperazine‐1‐ethanesulfonic acid (HEPES), 10 mm ethylene glycol tetraacetic acid (EGTA), and 4 mm MgSO4·7H20 (PHEM) buffer, at RT for ~10 min. After aspiration of the fixative, the cells were washed thoroughly with cold PBS and permeabilized using 0.1% Triton X‐100 for ~10 min, followed by additional washes with PBS. For visualization, fixed and permeabilized cells were stained with DAPI (10 μL of 50 μM) for 15 min and subsequently stained with PhenoVue Fluor 568‐Phalloidin (~0.4 nM, 20 μL) for 1 h. Each staining step was followed by washing with cold PBS. Imaging was performed using a Leica SP‐5 confocal microscope.

#### 2.5.3 Quantitative TNF‐α and IL‐6 assay

For determining the effect of 2DG‐D‐Rosi conjugates on neuroinflammation under in vitro conditions, the expression levels of tumor necrosis factor‐alpha (TNF‐α) and interleukin‐6 (IL‐6) were analyzed using an ELISA. The protocol was adapted from previously published literature with slight modifications.^22^, ^23^ Briefly, CATH.a neurons (5 × 10^4^ cells/well) were seeded into 96‐well plates and cultured overnight. The cells were pre‐treated with lipopolysaccharide (LPS; 1 μg/mL) and hydrogen peroxide (H_2_O_2_, 100 μM) and then incubated with 2DG‐D‐Rosi conjugates at varying concentrations (50, 100, and 250 μg/mL) for 24 h at 37°C in a CO_2_ incubator. Following the treatment, the consumed culture media was collected and centrifuged at 12,000 × *g* for 10 min to remove cellular debris. A 50 μL aliquot of the supernatant was added to ELISA plates pre‐coated with primary antibodies for TNF‐α and IL‐6, respectively, and incubated for approximately 2 h. The cytokine concentrations (pg/mL) were determined using standard calibration curves provided in the ELISA kit, adhering to the manufacturer's instructions. All experiments were performed in triplicates, with appropriate controls.

#### 2.5.4 Greiss reagent assay for nitric oxide estimation

The effect of dendrimer conjugates on reactive nitric oxide (NO) species was estimated using Greiss reagent‐based assay, following the published method with slight modification.^24^ For this, 5 × 10^4^ CATH.a cells/well were seeded in a 96‐well plate and grown in serum‐free media overnight. The media was changed, and the cells were treated with 2DG‐D‐Rosi dendrimer conjugates for 12 h at 37°C in a CO_2_ incubator. Following this, equal volumes (50 μL) of Greiss reagent (0.1%) and cell supernatant were mixed and incubated for 15 min for color change. Finally, absorbance was measured at 540 nm using a Thermo Scientific Multiskan Sky High Microplate Reader. The final NO concentrations were determined following the nitrate standard calibration curve. All the measurements were taken in triplicates with proper controls.

#### 2.5.5 Annexin V/propidium iodide assay

Apoptosis/necrosis was assessed in CATH.a cells using the FITC ApoScreen® Annexin V Apoptosis Kit. Cells were pre‐treated with LPS (1 μg/mL) and H₂O₂ (100 μM) for 6 h to mimic TBI‐like conditions, followed by 2DG‐D‐Rosi treatment for 24 h at 37°C, 5% CO_2_. After incubation, cells were harvested, washed twice with cold PBS, and resuspended in 100 μL of 1X Annexin Binding Buffer. Staining was performed with 10 μL Annexin V‐FITC and 10 μL propidium iodide (PI) for 15 min at 2–8°C in the dark, followed by dilution with 380 μL 1X Annexin Binding Buffer. Samples were analyzed on an Attune NxT Flow Cytometer using BL‐1 (530/30 nm) for Annexin V‐FITC and YL‐1 (585/16 nm) for PI. Controls included unstained, Annexin V‐only, and PI‐only samples. Density plots were generated to classify viable, early apoptotic, necrotic, and late apoptotic/necrotic cells. The experiment was performed in triplicate (*n* = 3), and data was analyzed using GraphPad Prism with statistical significance determined by a two‐tailed *t*‐test.

#### 2.5.6 Mitochondrial membrane potential (MMP) assay

The JC‐1 mitochondrial membrane potential (MMP) assay was used to assess 2DG‐D‐Rosi effects on CATH.a cells under TBI‐like conditions via flow cytometry and confocal microscopy. Cells were pre‐treated with LPS (1 μg/mL) and H_2_O_2_ (100 μM), followed by 2DG‐D‐Rosi treatment for 24 h at 37°C, 5% CO_2_. After incubation, cells were stained with 2 μM JC‐1 dye for 20 min at 37°C in the dark. For flow cytometry, cells were analyzed on an Attune NxT Flow Cytometer, with JC‐1 monomers (green, depolarized mitochondria) detected in BL‐1 (530/30 nm) and J‐aggregates (red, polarized mitochondria) in YL‐1 (585/16 nm). For confocal microscopy, cells were seeded on poly‐L‐lysine‐coated coverslips, stained with JC‐1, counterstained with DAPI, and imaged using a Leica SP‐5 Confocal Microscope (20× water immersion magnification). The experiment was conducted in triplicate (*n* = 3), and data were analyzed using GraphPad Prism, with statistical significance determined by a two‐tailed *t*‐test.

#### 2.5.7 Caspase activity assay

The caspase‐3/7 activity assay was conducted to assess 2DG‐D‐Rosi effects on apoptosis in CATH.a cells under TBI‐like conditions via flow cytometry and confocal microscopy. Cells were pre‐treated with LPS (1 μg/mL) and H_2_O_2_ (100 μM), followed by 2DG‐D‐Rosi treatment for 24 h (flow cytometry) or 48 h (confocal microscopy) at 37°C, 5% CO_2_. For flow cytometry, 1 × 10^5^ cells per sample were incubated with PhenoVue 505 caspase‐3/7 stain in fresh medium for 30 min, washed with PBS, and analyzed on an Attune NxT Flow Cytometer using BL‐1 (530/30 nm) for caspase activation detection. For confocal microscopy, 2 × 10^5^ cells per well were seeded on poly‐L‐lysine‐coated coverslips, stained with caspase‐3/7 reagent, fixed in 4% PFA, permeabilized with 0.1% Triton X‐100, and counterstained with DAPI. Coverslips were mounted with Vectashield Plus antifade medium and imaged using a Leica SP‐5 Confocal Microscope (20× water immersion lens). The experiment was conducted in triplicate (*n* = 3), and statistical significance was determined using GraphPad Prism with a two‐tailed *t*‐test.

#### 2.5.8 PPARγ expression analysis via immunofluorescence, flow cytometry, and dot blotting

PPARγ expression in CATH.a cells was assessed using flow cytometry and immunofluorescence following 2DG‐D‐Rosi treatment. Cells were pre‐treated with LPS (1 μg/mL) and H_2_O_2_ (100 μM), then incubated with 2DG‐D‐Rosi for 24 h (flow cytometry) or 48 h (immunofluorescence) at 37°C, 5% CO_2_. For flow cytometry, 1 × 10^5^ cells per sample were fixed in 4% PFA, permeabilized with 0.2% Triton X‐100, blocked with 3% BSA, and incubated overnight at 4°C with an anti‐PPARγ antibody. After washing, cells were incubated with PhenoVue Fluor‐488 IgG1 secondary antibody for 2 h at 37°C, washed again, and analyzed on an Attune NxT Flow Cytometer using BL‐1 (530/30 nm) for PPARγ detection.

For immunofluorescence, 5 × 10^4^ cells per well were grown on poly‐L‐lysine‐coated coverslips, treated, washed, and blocked with 3% BSA, followed by overnight incubation with an anti‐PPARγ antibody at 4°C on a 3D shaker. After washing, cells were incubated with PhenoVue Fluor‐488 IgG1 secondary antibody for 2 h at 37°C, washed, fixed, and permeabilized in 4% PFA and 0.2% Triton X‐100 for 10 min each, counterstained with DAPI, and mounted with VectaShield antifade medium. Imaging was performed using a Zeiss SP5 Confocal Microscope (20× water immersion lens). The experiment was conducted in triplicate (*n* = 3), and statistical significance was determined using GraphPad Prism with a two‐tailed *t*‐test.

Additionally, to validate and support our claims, the PPARγ expression in neurons was evaluated using the dot blotting technique following published reports with slight modifications.^25^ Aliquots of the isolated protein sample (5 μL) were spotted onto a nitrocellulose membrane and allowed to dry for 15 min. The membrane was blocked with 5% skimmed milk and 5% BSA in 1× Phosphate Buffered Saline with Tween 20 (PBST) for 2 h at RT. It was then probed with PPARγ primary antibody (1:1000) overnight at 4°C. After three washes with PBST, the membrane was incubated with the appropriate Horseradish Peroxidase (HRP)‐conjugated secondary antibody for 3 h at RT. The blot membranes were again washed three times and finally visualized using an ECL kit and imaged with the Bio‐Rad ChemiDoc gel documentation system.

### 2.6 In vivo studies

#### 2.6.1 Animals

Male and female C57BL/6 mice (2–3 months of age; Jackson Laboratory, Bar Harbor, ME) were in‐house bred as previously described.^13^, ^26^, ^27^, ^28^, ^29^ All animals were housed under ambient conditions (20–22°C, 40%–60% relative humidity, and a 12‐h light/dark cycle) with free access to food and water. All experiments followed the Guide for the Care and Use of Laboratory Animals, eighth edition, published by the National Research Council.^30^, ^31^ Experimental procedures were approved by the Institutional Animal Care and Use Committee (IACUC) of the University of Michigan (Protocol ID: PRO00010860).

#### 2.6.2 Impact acceleration model of TBI

On postnatal Days 20–21 (P20‐21), male (M) and female (F) animals (*n* = 84, 42 M/42 F) from the same litter were randomized into Sham (*n* = 20, 10 M/10 F) and TBI (*n* = 64, 32 M/32 F) groups using a random number generator. Randomization was stratified by sex. The TBI animals underwent the injury procedure as previously described.^13^, ^26^, ^27^, ^28^, ^29^ In brief, after being fully anesthetized, the animal was placed chest‐down on a platform with a trapdoor that supported the body weight of a mouse (approximately 7–10 g body weight) with little to no resistance or restraint upon impact. The animal's head was directly in the path of a falling weight. A weight (30 g) was held at 1.0 m above the platform and secured by a pin. The lab personnel pulled the pin, allowing the weight to fall vertically through a guide tube to strike the animal on the head in the midline between bregma and lambda (at approximately bregma −2.5 mm). Sham animals were anesthetized with 4% isoflurane without TBI impact. All animals were closely monitored postoperatively with weight and health surveillance recordings, as per IACUC guidelines.

#### 2.6.3 2DG‐D‐Rosi‐Cy5 co‐localization with neurons

Male and female TBI mice (*n* = 2 per sex) received a single intraperitoneal administration of fluorescent 2DG‐D‐Rosi‐Cy5 (50 mg/kg, 100 μL) at 6 h post‐injury and were euthanized at 24 h post‐injection. Brains were removed, postfixed in 10% formalin for 48 h, and then cryoprotected in 30% sucrose (in PBS). Coronal sections (20 μm, 1:6 series) were prepared on a cryostat (Leica Microsystems, IL, USA). Brain sections were incubated overnight at 4°C with primary antibodies. The primary antibodies included rabbit anti‐NeuN (a neuronal marker; 1:250; Cat# ab177487; Abcam, MA. USA), rabbit anti‐IBA1 (a microglial marker; 1:250; Cat# 019–19,741; FUJIFILM Wako Pure Chemical Corporation, CA. USA), rabbit anti‐GFAP (an astrocyte marker; 1:250; Cat# PA1‐10019; Thermo Fisher Scientific Inc., MA, USA), and rat anti‐CD31 (an endothelial marker; 1:250; Cat# MA1‐40074; Thermo Fisher Scientific Inc., MA, USA). Sections were subsequently washed and incubated with fluorescent secondary antibody (1:250; Life Technologies, MA, USA) for 2 h at RT. The slides were dried and cover‐slipped with fluorescent mounting medium with DAPI (Sigma‐Aldrich, MO, USA). Images were acquired using a Nikon Eclipse TS2R fluorescent microscope (Nikon, NY, USA).

#### 2.6.4 In vivo rosiglitazone/dendrimer‐rosiglitazone administration

Mice in the TBI group were randomized into TBI + vehicle (*n* = 20, 10 M/10 F), TBI + Rosi (*n* = 20, 10 M/10 F), and TBI + Dendrimer‐Rosi (TBI + 2DG‐D‐Rosi) (*n* = 20, 10 M/10 F) groups. Animals received a single intraperitoneal administration of free Rosi (2 mg/kg, 100 μL), dendrimer‐Rosi (containing 2 mg/kg Rosi, 100 μL) or vehicle (100 μL) at 6 h post‐injury. The mice from the sham group (*n* = 20, 10 M/10 F) did not receive any intervention. The efficacy of the drugs was evaluated at 24 h post‐drug administration.

#### 2.6.5 Body weight

Body weight was measured before injury (baseline) and at 1‐day post‐treatment. The changes in body weight were calculated as (Body weight)_change_ = (body weight)_1d_ − (body weight)_baseline_.

#### 2.6.6 Behavioral tests

All of the behavioral testing was performed between 7 a.m. and 6 p.m. Mice (*n* = 20 per group, 10 M/10 F) were habituated in the test room for at least 30 min before the behavioral tests.^26^, ^32^ The lab personnel were blinded to experimental groups.

##### Grip strength

Muscular strength was evaluated with a grip strength test using a grip strength meter (BIOSEB, FL, USA) before injury and at 1‐day post‐treatment.^13^ In brief, the grip strength meter was positioned horizontally and the animals were held by the tail and lowered towardthe apparatus. The animals were allowed to grab the metal grid and were then pulled backward in the horizontal plane. The force applied to the grid was recorded as the peak tension. Each animal underwent a grip strength test in three consecutive trials. The results were recorded and averaged for each animal. The change in the grip strength before and after injury was calculated as: (grip strength)_change_ = (grip strength) _24h_ – (grip strength) _baseline_.

##### Rotarod

Sensorimotor coordination, endurance, and fatigue resistance were evaluated with a touchscreen five station accelerating Panlab RotaRod for mouse (BIOSEB, FL, USA) before injury and at 1 day post‐treatment based on a published protocol.^13^, ^33^ Each animal was evaluated individually and underwent three consecutive trials (5 min each). In brief, during each trial, the animal was situated on a stationary rod for 10 s, and the rod was then set in motion with an accelerating speed of 3–30 rpm. If the animals fell from the rotarod, the trial was stopped automatically, and the latency to the first fall was recorded. If the animals did not fall during the 5‐min trial, the animal was removed from the rotarod, and a maximum score of 5 min was recorded. The average latency to the first fall from three trials was calculated for each animal. Finally, the change in the latency to the first fall before and after injury was calculated as: (Latency)_change_ = (latency)_24h_ − (latency)_baseline_. The complete data set is provided in Table S2.

##### Tail suspension test

The tail suspension test was performed at 1 day post‐treatment as previously described to evaluate depression‐like behaviors.^13^, ^33^ In brief, mice were suspended by taping their tails (three quarters of the distance from the base of the tail) to a vertical bar on a tail suspension stand. The animal tail was aligned with the bottom of the bar. The animals' activities were monitored continuously for 6 min. The time spent immobile over the 6 min period was quantified and compared among groups.

##### Light/dark box test

The light/dark box was purchased from Stoelting Co. (Wood Dale, IL, USA), and the test was performed as previously described.^13^, ^28^ The test was performed at 1 day post‐treatment. In brief, mice were placed in the middle of the brightly illuminated chamber and were allowed to move freely between the light and dark chambers for 10 min. Video recording was used to record animal behaviors. The time spent in the light chamber was recorded and analyzed.

##### Novel object recognition

The novel object recognition test was modified from published protocols^13^, ^32^, ^34^ and performed at 1 day post‐treatment. In brief, the test was composed of two trials. The mice explored two identical objects for 5 min during the “training trial” and then were placed back in their cages. After an inter‐trial break of 4 h, one of the previously exposed “old” objects was replaced with a new “novel” object, and the animals were allowed to explore these two objects for 5 min during the “probe trial” The discrimination index for the probed trial was used to analyze the cognitive outcomes. Discrimination index = time spent exploring the novel object/(time spent exploring the old object + time spent exploring the novel object) × 100%.

#### 2.6.7 Isolation of primary neurons

Animals were euthanized after completion of behavioral tests at 1‐day post‐treatment (*n* = 12 per group, 6 M/6 F). Primary neuron isolation was performed as previously described.^13^ In brief, brains were harvested and rinsed in Hank’s Balanced Salt Solution (HBSS) solution on ice. Meninges were removed, and the area of injury (approximately between bregma +2 mm and bregma −1 mm) in the TBI mice and the matching area in the sham mice were micro‐dissected as previously described.^26^ Brain tissues were transferred to Hyaluronic Acid‐Binding Globulin (HABG) solutions (60 mL HA, 1.2 mL B27, 0.176 mL Gln [0.5 mM final]), and minced (~0.5 mm) on ice. Brain tissues were incubated in HABG solution for 8 min at 30°C in a Boekel shaking incubator with a shaking speed of 90 rpm (Cole‐Parmer, Vernon Hills, IL, USA). Tissues were transferred to papain solutions (12 mg papain solids per 6 mL HA‐Ca, 0.015 mL Gln [0.5 mM final]) and incubated for 30 min at 30°C in a shaking incubator with a shaking speed of 90 rpm. Tissues were washed in HABG solution for 5 min at RT, triturated with a sterile pipette for 45 s, and kept at RT for 1 min. The supernatants were collected, and the trituration was repeated two times. The supernatants were combined and centrifuged in OptiPrep™ Density Gradient Medium at 800 × *g* for 15 min at 22°C, and the fractions of neurons were collected as previously described.^13^, ^35^ Cells were washed in HABG solutions and centrifuged at 200 × *g* for 2 min at 22°C. Supernatants were removed, and cells were washed in HBSS solution and centrifuged at 200 × *g* for 2 min at 22°C. Cell pellets were harvested for RNA isolation.

#### 2.6.8 RNA isolation and quantitative real‐time polymerase chain reaction

The mRNA expression of TNF‐α, IL‐1 beta (IL‐1β), Toll‐like receptor 4 (TLR4), NLR Family Pyrin Domain Containing 3 (NLRP3), IL‐13, and Fas was measured. Total RNA was extracted using TRIZOL (Sigma‐Aldrich, MO, USA), according to the manufacturer's instructions. RNA samples were quantified using the Nanodrop ND‐2000 Spectrophotometer (Thermo Fisher Scientific, MA, USA). Single‐stranded complementary DNA (cDNA) was reverse transcribed from RNA using the High‐Capacity cDNA Reverse Transcription Kit with RNase inhibitor (Thermo Fisher Scientific, MA, USA). Quantitative real‐time polymerase chain reaction (qPCR) was performed with iTaq(tm) Universal SYBR(R) Green Supermix (Bio‐Rad, CA, USA) with the CFX connect real‐time polymerase chain reaction (PCR) detection system (Bio‐Rad, CA, USA). Amplification conditions included 30 s at 95°C, 40 cycles at 95°C for 5 s, and 60°C for 30 s. Primers were custom designed (Table 1) and ordered from Integrated DNA Technology (Coralville, IA, USA). The comparative threshold cycle (Ct) method was used to assess differential gene expressions. The sham group was the reference group, and glyceraldehyde 3‐phosphate dehydrogenase (*Gapdh*) was the housekeeping gene. Gene expression levels for each sample were normalized to the expression level of *Gapdh* within a given sample (ΔCt); the differences between the sham and TBI groups were used to determine the ΔΔCt. The 2‐ΔΔCt gave the relative fold changes in gene expression.

**TABLE 1:** Primers for quantitative real‐time polymerase chain reaction.

Gene	Forward primer	Reverse primer
*Tnf‐α*	TCAGCCGATTTGCTATCTC ATA	AGTACTTGGGCAGATTGACCTC
*Il‐1β*	GGTGTGTGACGTTCCCATTA	ATTGAGGTGGAGAGCTTTCAG
*Tlr4*	GGGTATTTGACACCCTCCATAG	CAAGAGTGCTGAGGGAATACAG
*Nlrp3*	ACGTGTTCCAGAAGGAAGTG	GCCTCCTCTTCCAGCAAATA
*Il‐13*	GCTGAGCAACATCACACAAG	AATCCAGGGCTACACAGAAC
*Fas*	ACCATGCCAACCTGGTAAA	CATGTACTCCTTCCCTTCTGTG
*Gapdh*	AACAGCAACTCCCACTCTTC	CCTGTTGCTGTAGCCGTATT

#### 2.6.9 Immunohistochemistry

Animals were euthanized after completion of behavioral tests at 1‐day post‐treatment (*n* = 8 per group, 4 M/4 F), and transcardially perfused with PBS. Brains were removed, postfixed in 10% formalin for 48 h, and then cryoprotected in 30% sucrose (in PBS). Coronal sections (20 μm, 1:6 series) were prepared on a cryostat (Leica Microsystems, IL, USA). Brain sections were washed 3 times in PBS for 5 min each, incubated in 0.001% Fluoro‐Jade C (FJC) (Cat# TR‐160‐FJC, Biosensis, CA, USA) for 10 min, washed six times in PBS for 15 min each, and incubated with rabbit anti‐NeuN (1:250; Cat# ab177487; Abcam, MA, USA). Sections were subsequently washed and incubated with fluorescent secondary antibodies (1:250; Life Technologies, MA, USA) for 2 h at RT. The slides were dried and cover‐slipped with fluorescent mounting medium with DAPI (Sigma‐Aldrich, MO, USA).

#### 2.6.10 Histology quantification

Images (40×, 5 images/animal) were randomly acquired from the cortex area (mainly primary motor cortex and primary somatosensory cortex) in the injured brain regions (approximately between bregma +1 mm and bregma −0.5 mm; Figure S25A,B) using the Nikon Eclipse TS2R fluorescent microscope (Nikon, NY, USA), and the camera settings were kept the same for all the experimental groups. All slides and images were coded, and the analysis was performed with personnel blinded to the experiments. The co‐localization of FJC^+^NeuN^+^ cells was analyzed using “Coloc 2” function in Fiji ImageJ (National Institutes of Health [NIH]) as previously described.^28^ In brief, the original images were split into separate channels (FJC: green; NeuN: red) and were converted to 8‐bit. Background subtraction was performed using Fiji ImageJ following the manufacturer's instructions. The co‐localization was analyzed using Kendall's Tau Rank Correlation test, and Costes' randomization was set at 10. To ensure the accuracy of analysis, data were manually checked with co‐localization analysis as described previously.^26^, ^29^, ^34^, ^36^ The color images presented in the results section were processed by adjusting the brightness and contrast using Fiji ImageJ (NIH) following the manufacturer's instructions.

#### 2.6.11 Statistical analysis

Data was analyzed using GraphPad Prism 6 (Version 6.04; CA, USA). All data were presented as mean ± Standard Error of the Mean (SEM). Kolmogorov–Smirnov normality test was used for normality measurement. One‐way and two‐way Analysis of Variance (ANOVA) and Bonferroni post hoc test were used for multiple group comparisons. Statistical significance was set at *p* < 0.05 for all analyses.

## 3 RESULTS AND DISCUSSION

### 3.1 Synthesis and physicochemical characterization of 2DG‐D‐Rosi conjugate

**FIGURE 1:**
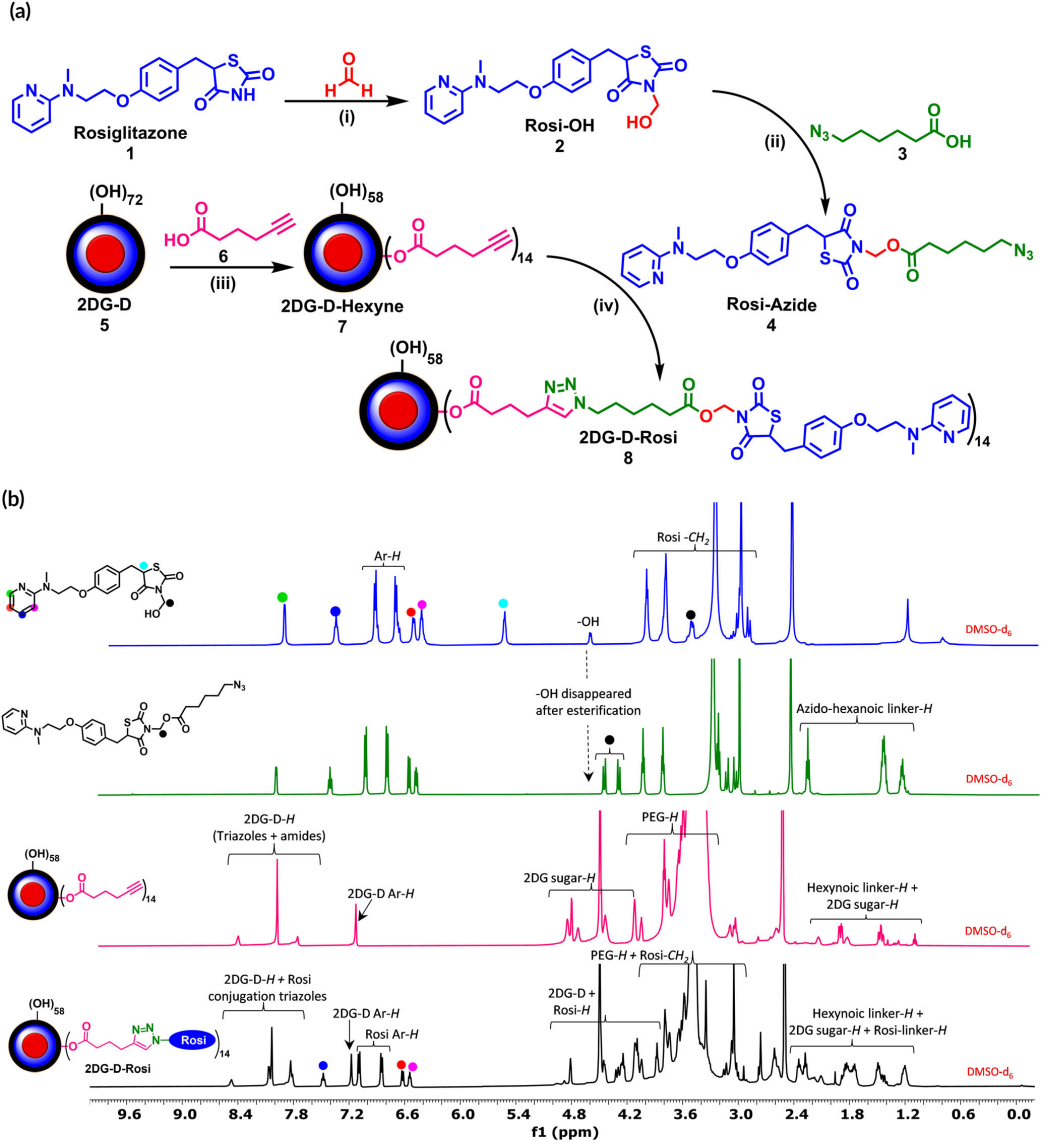
(a) Synthesis of rosiglitazone (Rosi) intermediates and 2‐deoxyglucose (2DG‐D)‐Rosi. *Reagents and conditions*: (i) triethylamine, anhy. DMF, room temperature (RT), 10 h; (ii) 1‐[3‐(dimethylamino)propyl]‐3‐ethylcarbodiimide (EDC) • HCl, 4‐(dimethylamino)pyridine (DMAP), anhy. DMF, RT, 4 h; (iii) EDC • HCl, DMAP, dry DMF, RT, 24 h; (iv) CuBr, PMDETA, THPTA, anhy. DMF, RT, 10 h; (b) ^1^H NMR characterization of Rosi intermediates and 2DG‐D‐Rosi showing characteristic protons signals.

The synthesis of the 2DG‐D‐Rosi was initiated by the modification of Rosi with an azide‐terminating linker for subsequent conjugation on alkyne‐terminating 2DG‐D, utilizing copper‐catalyzed azide‐alkyne click (CuAAC) chemistry. More specifically, Rosi was first reacted with formaldehyde to generate Rosi‐OH (**2**), as illustrated in Figure 1a. The ^1^H NMR spectra of Rosi‐OH displayed signals between *δ* 3.67 and 3.57 ppm, confirming the presence of the ‐CH_2_OH group attached to the nitrogen atom (Figures 1b and S1). Subsequently, Rosi‐OH (**2**) was converted into Rosi‐Azide (**4**) by the attachment of clickable linker 6‐azido‐hexanoic acid (**3**) using EDC‐DMAP as the coupling agent. The ^1^H NMR spectrum of Rosi‐Azide (**4**) revealed characteristic signals of the aliphatic chain from azido‐hexanoic acid, with proton peaks observed between *δ* 2.32 and 1.28 ppm. Additionally, the two protons (‐CH_2_) attached to the nitrogen of the thiazolidinedione ring appeared as distinct doublets at *δ* 4.52 and *δ* 4.37 ppm (Figures 1b and S5).

**FIGURE 2:**
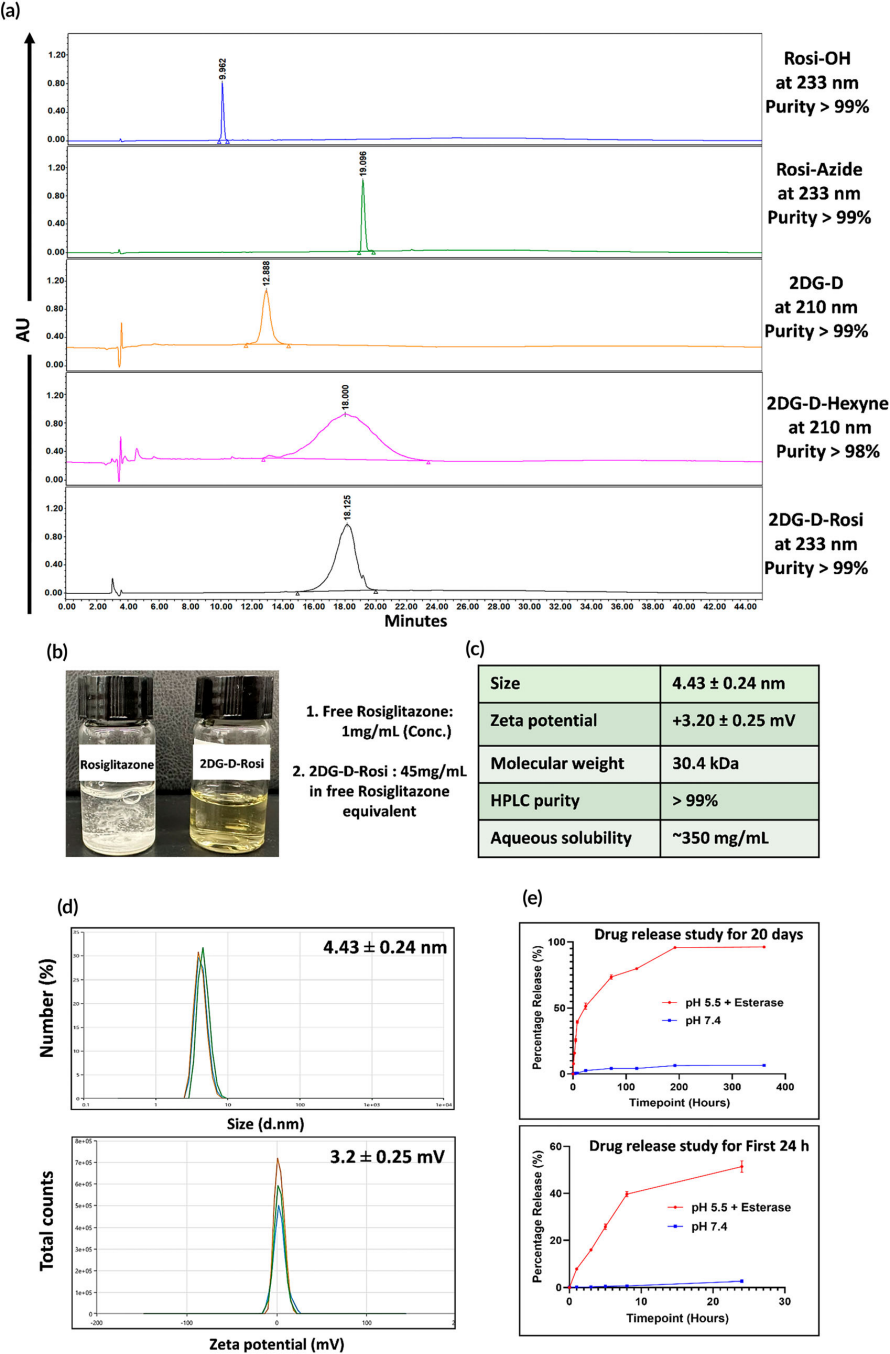
(a) High‐performance liquid chromatography traces of rosiglitazone (Rosi) intermediates, 2‐deoxyglucose (2DG‐D), 2DG‐D‐hexyne, and 2DG‐D‐Rosi, confirming successful conjugation. (b) A significant enhancement in the water solubility of Rosi upon conjugation with 2DG‐D. (c) Physicochemical properties of 2DG‐D‐Rosi. (d) Size distribution and zeta potential of 2DG‐D‐Rosi. (e) In vitro drug release study of 2DG‐D‐Rosi over 20 days, demonstrating sustained release of Rosi.

Next, we synthesized 2DG‐D‐Hexyne (**7**), functionalizing 2DG‐D (**5**) with hexyne groups through esterification (Figure 1). Hexynoic acid (**6**) was reacted with the hydroxyl groups on the surface of the dendrimer using EDC and DMAP as coupling reagents, resulting in the formation of 2DG‐D‐hexyne (**7**). The number of alkyne groups on the dendrimer can be tailored to achieve the desired drug loading capacity. The formation of 2DG‐D‐hexyne was confirmed using HPLC, which demonstrated a shift in retention time compared to the unfunctionalized dendrimer, indicating the formation of product (Figure 2a). Additionally, ^1^H NMR spectra revealed characteristic aliphatic proton signals in the range of *δ* 2.32–1.28 ppm, confirming the presence of the methylene protons of the hexynoic acid linker (Figures 1b and S8). The alkyne groups on the dendrimer were then conjugated with Rosi‐Azide (**4**) via CuAAC. This reaction, performed in an anhydrous environment using CuBr and PMDETA in dry DMF, yielded 2DG‐D‐Rosi (**8**) with greater efficiency compared to aqueous conditions using copper sulfate and NaAs, likely attributed to the enhanced stability of the Rosi‐Azide in the absence of water. The formation of the triazole rings from the click reaction was confirmed by the appearance of new peaks in the ^1^H NMR spectra, observed between *δ* 8.02 and 7.82 ppm, which are characteristic of triazole protons. Additionally, the aromatic proton signals from Rosi at *δ* 7.08 and 6.84 ppm further validated the successful attachment of approximately ~14 drug molecules to the dendrimer (Figures 1b, S11, and S12). The purity of the resulting 2DG‐D‐Rosi was ~99% (Figure S14), as determined by HPLC analysis, which also showed a significant shift in retention time from 18.0 min (2DG‐D‐hexyne) to 18.12 min (2DG‐D‐Rosi), further confirming successful conjugation (Figure 2a). The drug loading was assessed by the NMR integration method, suggesting a drug loading of 12%–13% by weight, corresponding to the attachment of ~14 Rosi molecules per dendrimer. Detailed characterization of intermediates and the final 2DG‐D‐Rosi conjugate are carried out via NMR and mass spectroscopy techniques, and the purity was determined using HPLC (Figures S1–S15).

Rosi exhibits significant challenges in terms of solubility and is sparingly soluble in water. This limited solubility restricts bioavailability and therapeutic applications. However, conjugation with the 2DG‐D dendrimer markedly enhanced the solubility of Rosi (Figure 2b). The physicochemical properties for 2DG‐D‐Rosi are detailed in Figure 2c. The zeta potential of 2DG‐D‐Rosi was determined to be nearly neutral (3.2 ± 0.25 mV), and the size distribution was measured at 4.43 ± 0.24 nm (Figure 2d), characteristics that facilitate extravasation through leaky blood vessels at the brain injury site and efficient movement through the brain tissue parenchyma.^13^, ^37^ Additionally, the small size of the conjugate also supports renal clearance from off‐target organs, thereby ensuring minimizing systemic toxicity.

### 3.2 In vitro drug release and shelf stability studies

Rosi was conjugated to 2DG‐D through an enzyme‐cleavable ester linkage for intracellular drug release. The drug release behavior of 2DG‐D‐Rosi was evaluated under two biologically relevant conditions to provide insight into its potential performance in vivo. First, drug release was studied at physiological pH (7.4) in PBS at 37°C, which simulates plasma conditions. The conjugate demonstrated stability in plasma conditions with less than 5% release over 2 weeks, ensuring minimal premature drug leakage during transport to the target site. Second, the drug release was evaluated under acidic conditions (pH 5.5) in the presence of esterase from porcine liver to mimic the intracellular endosomal/lysosomal environment. The acidic pH reflects the characteristic environment of endosomes/lysosomes, while esterase was included to simulate enzymatic activity that could trigger drug release upon cellular uptake. At intracellular conditions, ~50% of the drug was released within 2–3 days, and ~80% was released over 12 days, demonstrating a sustained intracellular release profile of the conjugate (Figure 2e). It is worth noting that the aim of this experiment is to model the intracellular drug release pathway relevant to dendrimer uptake via endocytosis, rather than the complex extracellular brain environment post‐injury.

Next, the shelf life stability of 2DG‐D‐Rosi formulation in PBS was evaluated under two storage conditions: at 25°C (RT) and 4°C (Figures S16 and S17). HPLC data demonstrated exceptional stability, with the purity of 2DG‐D‐Rosi remaining unchanged over 28 days, varying negligibly between 99.44% and 98.09%, with no significant shifts in retention time confirming the structural integrity of the formulation under these conditions. The lack of notable changes in retention time strongly suggests that the conjugated Rosi remained stably bound to the dendrimer, with no detectable release of the free drug during storage. The stability of 2DG‐D‐Rosi under these storage conditions has significant implications for its potential clinical applications. The ability to retain its purity and structural integrity for extended periods ensures reliable handling, transport, and storage, which are critical for transitioning from preclinical to clinical settings. Furthermore, the negligible degradation or release of the free drug minimizes concerns regarding toxicity or reduced efficacy due to drug release during storage.

To investigate the in vitro and in vivo neuronal uptake of 2DG‐D‐Rosi via confocal microscopy, we attached a near infra‐red fluorescent tag Cy5 to develop 2DG‐D‐Rosi‐Cy5 (11; Figure S18). The synthesis began by conjugating Cy5 to 2DG‐D through the modification of its surface hydroxyl groups with 5‐hexynoic acid using EDC‐DMAP, resulting in 2DG‐D‐hexyne (**9**), as illustrated in Figure S18A. Subsequently, 2DG‐D‐hexyne (**9**) was reacted with Cy5 azide via CuAAC to form 2DG‐D‐Cy5 (**10**). Characterization by ^1^H NMR confirmed the incorporation of around two to three Cy5 molecules per dendrimer (Figure S19). Next, 2DG‐D‐Rosi‐Cy5 (**11**) was synthesized by reacting 2DG‐D‐Cy5 (**10**) with Rosi‐Azide (**4**) in the presence of anhydrous CuBr, again utilizing the CuAAC reaction (Figure S18A). The reaction progress was monitored by HPLC, and once Rosi‐Azide was fully consumed, the reaction was stopped, and the product was purified using size exclusion chromatography. The successful formation of 2DG‐D‐Rosi‐Cy5 was confirmed by ^1^H NMR, which exhibited proton signals from the pyridyl ring of Rosi at *δ* 6.29 ppm confirming the successful attachment of seven to eight Rosi molecules (Figures S18B, S21, and S22). A distinct shift in retention time was observed between 2DG‐D‐Cy5 (17.34 min; Figure S18C) and the synthesized 2DG‐D‐Rosi‐Cy5 (17.91 min; Figure S20), further validating the conjugation of Rosi.

### 3.3 Role of glucose transporters (GLUT) in the neuronal uptake of 2DG‐D‐Rosi conjugate

**FIGURE 3:**
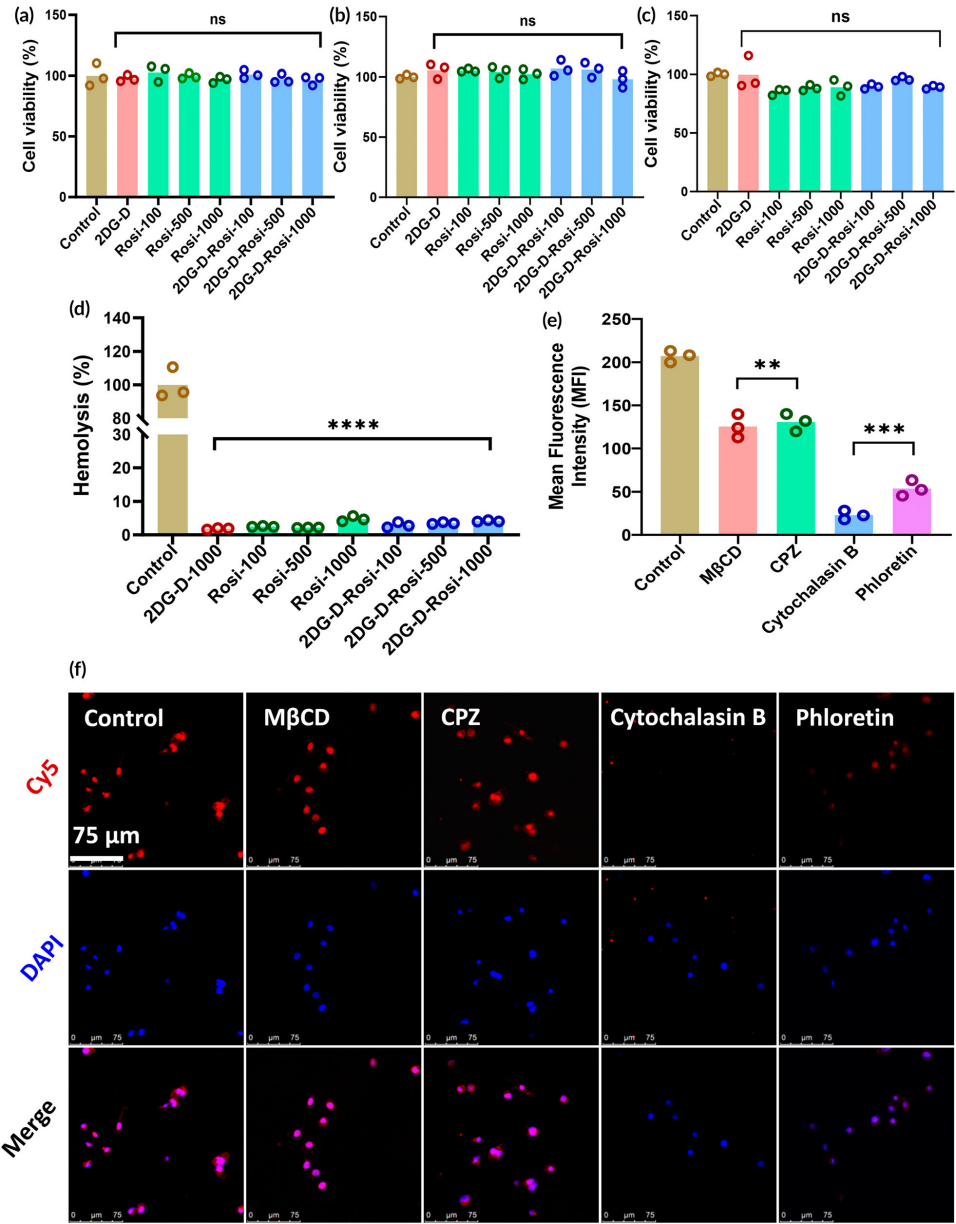
Evaluation of biocompatibility and neuronal uptake of dendrimers. (a–d) Cytocompatibility assay of 2‐deoxyglucose‐rosiglitazone (2DG‐D‐Rosi) conjugates at different concentrations with (a) CATH.a and (b) human umbilical vein endothelial cells, and (c) EOC 20 cells, and (d) hemocompatibility index of 2DG‐D‐Rosi dendrimer conjugates. (e and f) Confocal microscopy‐based (e) quantitative and (f) qualitative uptake of 2DG‐D‐Rosi dendrimers in CATH.a cells in the presence of trafficking inhibitors. The scale bar shown in the (f) is 75 μm. For cytocompatibility and hemocompatibility studies, the statistical significance was calculated using ordinary one‐way ANOVA, ****p < 0.0001, ***p < 0.001, **p < 0.01 ns ‐ nonsignificant. CPZ, chlorpromazine; Cy5, cyanine 5; DAPI, 4′,6‐diamidino‐2‐phenylindole; MβCD; methyl‐β‐cyclodextrin.

Before using 2DG‐D‐Rosi in either in vitro or in vivo experiments, we assessed the compatibility of the dendrimer conjugates with RBCs and three representative cell lines, viz., CATH.a neurons, human umbilical vein endothelial cells (HUVECs), and EOC 20 microglial cells. An MTT cell viability assay was conducted to evaluate the cytotoxicity of 2DG‐D‐Rosi on these cell lines. No toxic effects were observed at all tested concentrations of 2DG‐D, Rosi, or the 2DG‐D‐Rosi conjugate (Figure 3a–c). Additionally, no significant hemolysis was observed in RBCs treated with the 2DG‐D‐Rosi conjugate at all tested concentrations, with an overall hemolysis percentage below 5%, suggesting excellent compatibility with RBCs (Figure 3d) and confirming that 2DG‐D‐Rosi conjugates were safe to use further for carrying out in vitro/in vivo neuronal uptake and efficacy studies.

Previous reports have shown that glucose transporters (GLUTs), especially GLUT3, are key protein transporters that mediate the uptake of glucose and glucose‐based nanocarriers.^13^, ^38^, ^39^ To investigate the internalization mechanism of the 2DG‐D‐Rosi‐Cy5 conjugate, we conducted uptake studies in the presence of trafficking inhibitors. The results revealed no significant reduction in the Cy5 (red fluorescence) signal when cells were treated with MβCD or CPZ, compared to the untreated control, indicating that caveolae‐ and clathrin‐dependent endocytosis were not significantly involved in the uptake of the 2DG‐D‐Rosi‐Cy5 dendrimer by neuronal cells (Figure 3e,f). In contrast, treatment with cytochalasin B and phloretin, both GLUT inhibitors, led to a marked decrease in the uptake of 2DG‐D‐Rosi‐Cy5, with cytochalasin B causing a more substantial reduction in the Cy5 signal compared to phloretin. This suggests a predominant role of GLUT transporters in the uptake of 2DG‐D‐Rosi‐Cy5. Quantitative analysis using ImageJ further confirmed these observations, with the mean fluorescence intensity (MFI) for MβCD‐treated cells (0.9‐fold, *p* < 0.005) being similar to the untreated control (one‐fold), while the MFI was reduced to 0.86‐fold (*p* < 0.006), 0.11‐fold (*p* < 0.0001), and 0.26‐fold (*p* < 0.0003) for CPZ, cytochalasin B, and phloretin inhibitor‐treated cells, respectively. These findings collectively indicate the role of GLUT transporters in the uptake of 2DG‐D‐Rosi (Figure 3e,f). Several reports show enhanced in vivo uptake of glycosylated nanoparticles in cells over‐expressing GLUT receptors.^39^, ^40^, ^41^ However, we do not claim that the entire dendrimer conjugate is directly translocated through the GLUT transporter due to its small size. Instead, we hypothesize that the 2DG moiety on the dendrimer surface facilitates a high‐affinity interaction with the extracellular glucose binding site of GLUT transporters, leading to an atypical internalization process that may resemble transporter‐facilitated endocytosis or other mechanisms triggered by transporter engagement. There is growing evidence in the literature that some membrane transporters, when engaged by multivalent ligands, can recruit scaffolding proteins or initiate non‐canonical uptake pathways, including macropinocytosis‐like processes or clathrin/caveolin‐independent routes.^42^, ^43^, ^44^ Additional mechanistic studies are needed to definitively establish the nature of this interaction and internalization route.

### 3.4 2DG‐D‐Rosi attenuates inflammatory response and provides neuroprotection in LPS:H_2_O_2_ induced CATH.a cells

**FIGURE 4:**
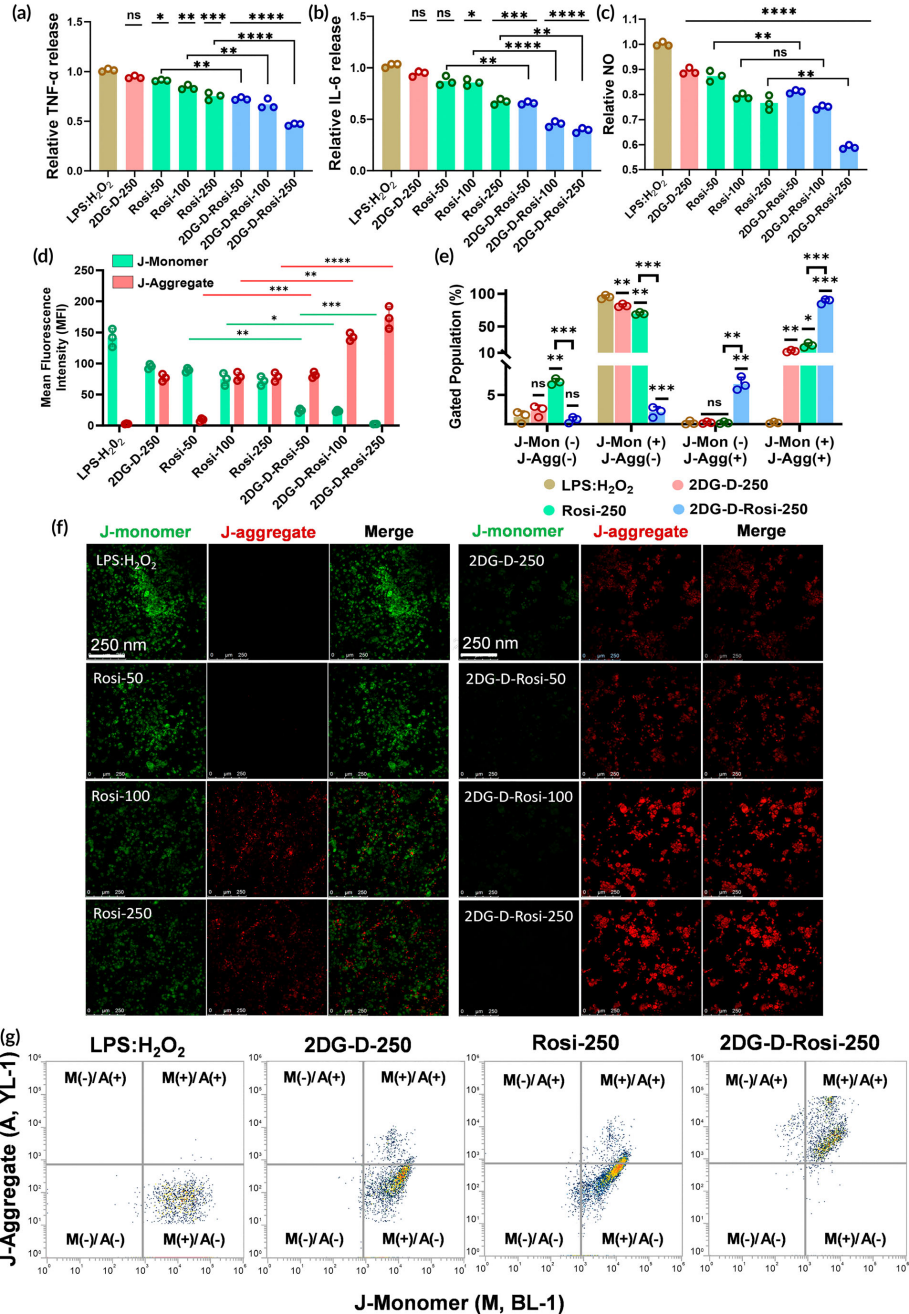
Effect of 2‐deoxyglucose‐rosiglitazone (2DG‐D‐Rosi) on regulating inflammatory response and mitochondrial health. Change in (a) tumor necrosis factor‐alpha (TNF‐α), (b) interleukin‐6 (IL‐6) and (c) nitric oxide (NO) levels after Rosi or 2DG‐D‐Rosi treatment in lipopolysaccharide:hydrogen peroxide (LPS:H_2_O_2_) induced CATH.a Cells. (d) Quantitative ImageJ bar gaph dipicting mean fluorescence intensity of j‐monomer/j‐aggregate in Rosi or 2DG‐D‐Rosi‐treated CATH.a cells. (e) Quantitative analysis showing percentage cells with polarized and depolarized mitochondria. (f) Confocal microscopy‐based analysis of neuronal mitochondrial membrane potential showing the effect of Rosi or 2DG‐D‐Rosi on mitochondrial health, as indicated by change in j‐monomer/aggregate fluorescence signal, (g) dot plots showing j‐monomer/j‐aggregate stained cell populations. The scale bar is 250 μm and the statistical significance was calculated using one‐way ANOVA, ****p < 0.0001; ***p < 0.001; **p < 0.01; *p < 0.05; ns, nonsignificant.

Shortly after TBI, there is a rapid surge in pro‐inflammatory mediators in the brain, particularly TNF‐α.^45^ Persistent elevation of TNF‐α in injured brain tissue has been shown to exacerbate trauma by increasing oxidative stress, disrupting blood–brain barrier (BBB), and amplifying inflammation, which further intensifies the severity of the traumatic injury.^46^, ^47^ Furthermore, the elevated levels of IL‐6 have also been observed to worsen the TBI condition by disruption of BBB and causing cerebral edema.^48^, ^49^ Hence, to explore the impact of 2DG‐D‐Rosi on the TNF‐α and IL‐6 levels in the CATH.a neuronal cells, we induced TBI‐like conditions using LPS:H_2_O_2_ and treated the cells with 2DG‐D‐Rosi dendrimer conjugates. The results revealed a dose‐dependent decrease in TNF‐α and IL‐6 levels in the treated cells compared to the untreated control group. Specifically, after treatment, TNF‐α levels showed a slight reduction from one‐fold in the control (LPS:H_2_O_2_) group to 0.94‐fold in empty 2DG‐D at 250 μg/mL (2DG‐D‐250). At concentrations of 100, 250, and 500 μg/mL, TNF‐α levels significantly decreased to 0.91, 0.85, and 0.77‐fold for Rosi, and 0.73, 0.68, and 0.47‐fold for 2DG‐D‐Rosi, respectively, though the decrease was much more pronounced in the 2DG‐D‐Rosi group than in the only Rosi‐treated group (Figure 4a). Similarly, IL‐6 levels followed a comparable trend, with the control group at ~1‐fold, while 2DG‐D‐250 reduced IL‐6 to 0.96‐fold. Rosi treatment led to reductions of 0.88, 0.86, and 0.67‐fold, while 2DG‐D‐Rosi exhibited the most significant decreases, reducing IL‐6 release to 0.66‐, 0.45‐, and 0.39‐fold at 50, 100, and 250 μg/mL, respectively (Figure 4b). These findings demonstrate a dose‐dependent reduction in the inflammatory response in neurons following treatment with 2DG‐D‐Rosi dendrimer conjugates, highlighting the superior efficacy of 2DG‐D‐Rosi in mitigating neuroinflammation compared to Rosi.

NO, synthesized by nitric oxide synthase (NOS) in neurons, plays a critical role in neuroprotection under controlled physiological conditions. However, in TBI conditions, heightened neuroinflammation leads to the excessive production of NO, which has deleterious effects, including neuronal damage and cell death.^50^, ^51^ To address this, we investigated the impact of 2DG‐D‐Rosi dendrimer conjugates on NO generation in neurons. Our quantitative analysis revealed that treatment with 2DG‐D‐Rosi resulted in a dose‐dependent reduction in NO production across all treated cells. Compared to the LPS:H_2_O_2_, NO generation was significantly decreased in all experimental groups. Notably, a comparison between free Rosi and 2DG‐D‐Rosi demonstrated a more pronounced difference in reduction in NO levels for 2DG‐D‐Rosi. Specifically, the relative NO concentrations for Rosi and 2DG‐D‐Rosi were 0.89‐ and 0.82‐fold (*p* < 0.01), 0.78‐ and 0.76‐fold (not significant), and 0.76‐ and 0.58‐fold (*p* < 0.01) at 50, 100, and 250 μg/mL, respectively (Figure 4c). These findings highlight the superior efficacy of 2DG‐D‐Rosi in modulating NO levels compared to Rosi alone. The significant reduction in NO generation observed with 2DG‐D‐Rosi can likely be attributed to the enhanced intracellular delivery of 2DG‐D‐Rosi.

### 3.5 2DG‐D‐Rosi protects neurons from mitochondrial dysfunction by maintaining mitochondrial membrane potential

Following TBI, there are several pathophysiological and biochemical changes occurring at the trauma site, which result in the cascade of secondary injury occurring hours or days after the insult. These changes further result in the activation of cellular processes like increased reactive oxygen species (ROS) generation,^52^ post‐traumatic neuroinflammation,^15^ mitochondrial dysfunction^53^ etc., which ultimately lead to cell death.^49^ To evaluate the impact of 2DG‐D‐Rosi on mitochondrial homeostasis, we performed JC‐1 dye‐based confocal microscopy and flow cytometry studies. Following the induction of TBI‐like conditions and subsequent dendrimer treatment, JC‐1 dye was used to assess changes in MMP in the presence of 2DG‐D‐Rosi dendrimer conjugates. Quantitative ImageJ analysis of the confocal micrographs revealed that untreated control cells exhibited strong green fluorescence, indicative of J‐monomer formation due to mitochondrial dysfunction and depolarized membranes (Figure 4d,f). In contrast, treatment with Rosi and 2DG‐D‐Rosi at concentrations of 50, 100, and 250 μg/mL demonstrated a dose‐dependent decrease in the MFI of J‐monomers. In the control group, the MFI was ~141, which dropped to ~89, 74, and 72 with Rosi treatment, and to 24, 23, and 2.3 with 2DG‐D‐Rosi treatment. Concurrently, a significant increase in red fluorescence intensity was observed, indicating enhanced mitochondrial polarization and improved membrane potential, suggesting restored mitochondrial health. Notably, cells treated with 2DG‐D‐Rosi conjugates exhibited a substantial increase in red fluorescence (J‐aggregates), where the MFI increased from ~2.7 in the LPS:H_2_O_2_ group to ~78.1 and ~174 at 250 μg/mL for Rosi and 2DG‐D‐Rosi, respectively, highlighting superior restoration of MMP and mitochondrial function by 2DG‐D‐Rosi in comparison to Rosi (Figures 4d,f and S23, Table S1). Flow cytometry analysis further confirmed these findings, showing that LPS + H_2_O_2_ treatment resulted in approximately 95.13% depolarized mitochondria (J‐Mon+/J‐Agg‐), with minimal polarized (~0.23%) or healthy (~0.24%) mitochondria. Treatment with 2DG‐D‐250 and Rosi‐250 significantly reduced depolarized mitochondria (~82.00% and ~69.73%, respectively, *p* < 0.01), while increasing healthy mitochondria (~12.61%, *p* < 0.01 and ~21.54%, *p* < 0.01). Notably, the treatment with 2DG‐D‐Rosi drastically reduced depolarized mitochondria (~2.09%, *p* < 0.001) while significantly increasing both polarized (~6.89%, *p* < 0.01) and healthy mitochondria (~88.56%, *p* < 0.001), demonstrating its superior mitochondrial protective effects in the neuronal cells compared to free drug (Figure 4e,g).

Overall, these findings confirm that 2DG‐D‐Rosi dendrimer conjugates significantly improve mitochondrial health and exhibit enhanced efficiency in neuronal rescue by mitigating LPS:H_2_O_2_‐induced mitochondrial damage.

### 3.6 2DG‐D‐Rosi attenuates apoptosis and necrosis in CATH.a cells under TBI‐like conditions

**FIGURE 5:**
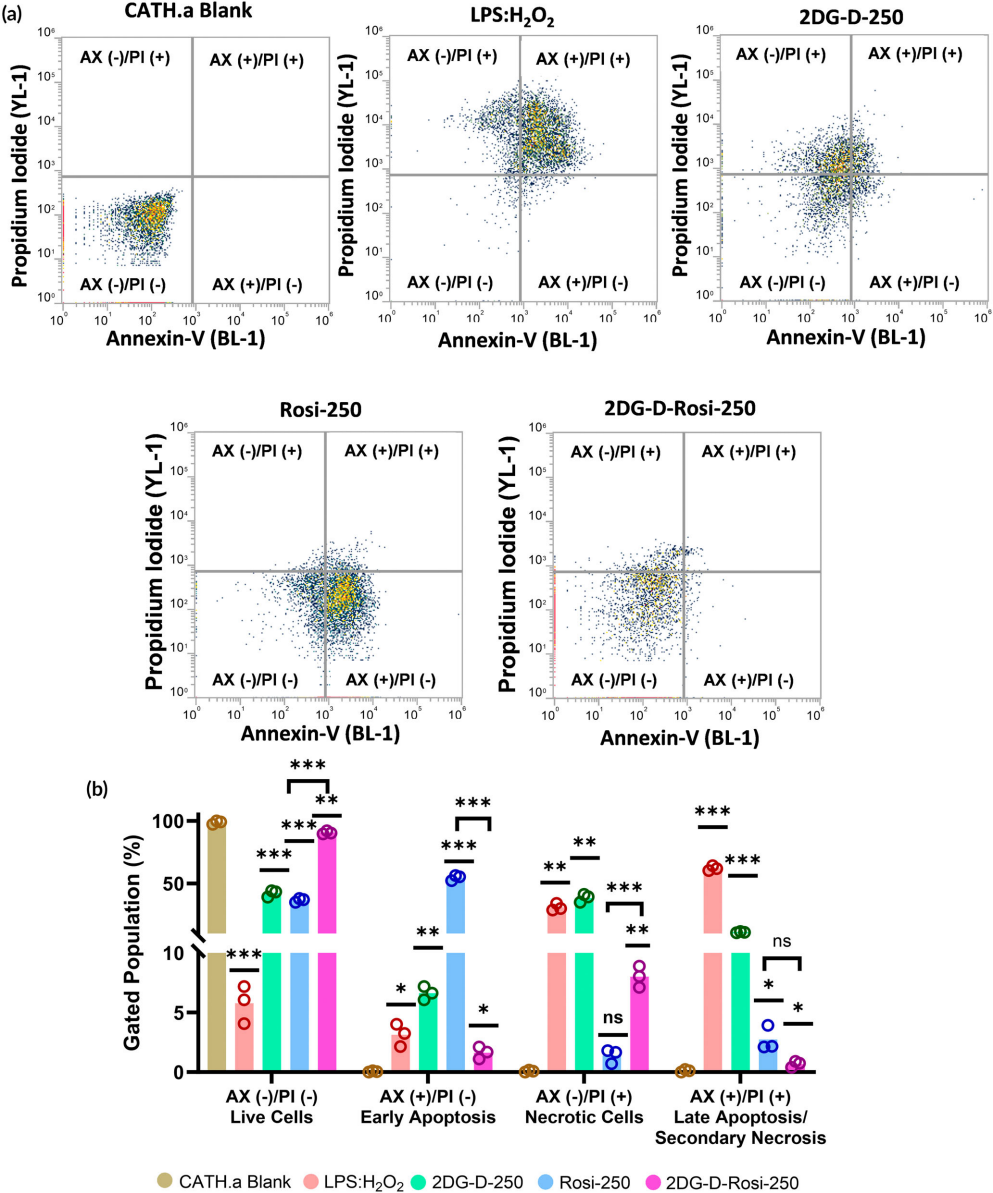
2‐Deoxyglucose‐rosiglitazone (2DG‐D‐Rosi) attenuates apoptosis and necrosis in CATH.a cells. (a) Apoptosis/necrosis analysis performed via flow cytometry in treated CATH.a neuronal cell. The dot plots show Annexin V (Alexa Fluor 488, YL‐1) and propidium iodide (PI; BL‐1) staining. The quadrants indicate live cells (Annexin V−/PI−), early apoptotic cells (Annexin V+/PI−), late apoptotic/necrotic cells (Annexin V+/PI+), and necrotic cells (Annexin V−/PI+). (b) Quantitative analysis showing percentage of cells in apoptotic/necrotic stage. For apoptosis/necrosis assay, the *p*‐values were calculated between the blank CATH.a cells and the treatments, as well as between Rosi and 2DG‐D‐Rosi using ordinary one‐way/two‐way ANOVA with, ***p < 0.001; **p < 0.01; *p < 0.05; ns‐nonsignificant; H_2_O_2_, hydrogen peroxide; LPS, lipopolysaccharide.

Apoptosis plays a critical role in neuronal injury and repair, particularly under conditions of oxidative and inflammatory stress. To assess the cytotoxic effects of 2DG‐D‐Rosi in CATH.a cells, we performed an Annexin V‐FITC/PI apoptosis assay using flow cytometry (Figure 5a,b). Untreated cells exhibited high viability (~98.7% AX‐V−/PI−) with minimal apoptosis/necrosis (~0.09% AX‐V−/PI+), confirming a baseline low apoptotic rate. In contrast, LPS:H_2_O_2_ treatment significantly reduced cell viability (~5.78%, *p* < 0.001) and induced a sharp increase in necrosis (~31.07%, *p* < 0.001) and late apoptosis (~62.1%, *p* < 0.001), confirming severe oxidative stress‐induced cell death. 2DG‐D alone at 250 μg/mL displayed modest protective effects, increasing viability (~42.2%, *p* < 0.001) while reducing necrosis (~38.56%) compared to LPS:H_2_O_2_, but still exhibited notable apoptotic activity (~6.62% AX‐V+/PI−). Rosi alone at 250 μg/mL resulted in a significant shift toward apoptosis (~54.6%, *p* < 0.001), but maintained a small necrotic population (~1.67%, ns), similar to untreated CATH.a cells, indicating partial protection. Notably, 2DG‐D‐Rosi treatment resulted in the highest viability (~90.63%, *p* < 0.001) with significantly reduced apoptosis (~1.63%) and necrosis (~8.01%), suggesting its neuroprotective potential in mitigating TBI‐induced oxidative damage. These results indicate that 2DG‐D‐Rosi effectively prevents apoptosis and necrosis, likely by stabilizing mitochondrial function and reducing oxidative stress. The significant differences observed across conditions suggest a mechanistic role in mitigating apoptotic and necrotic cell death pathways by 2DG‐D‐Rosi.

### 3.7 2DG‐D‐Rosi conjugates alleviates caspase activity in neurons

**FIGURE 6:**
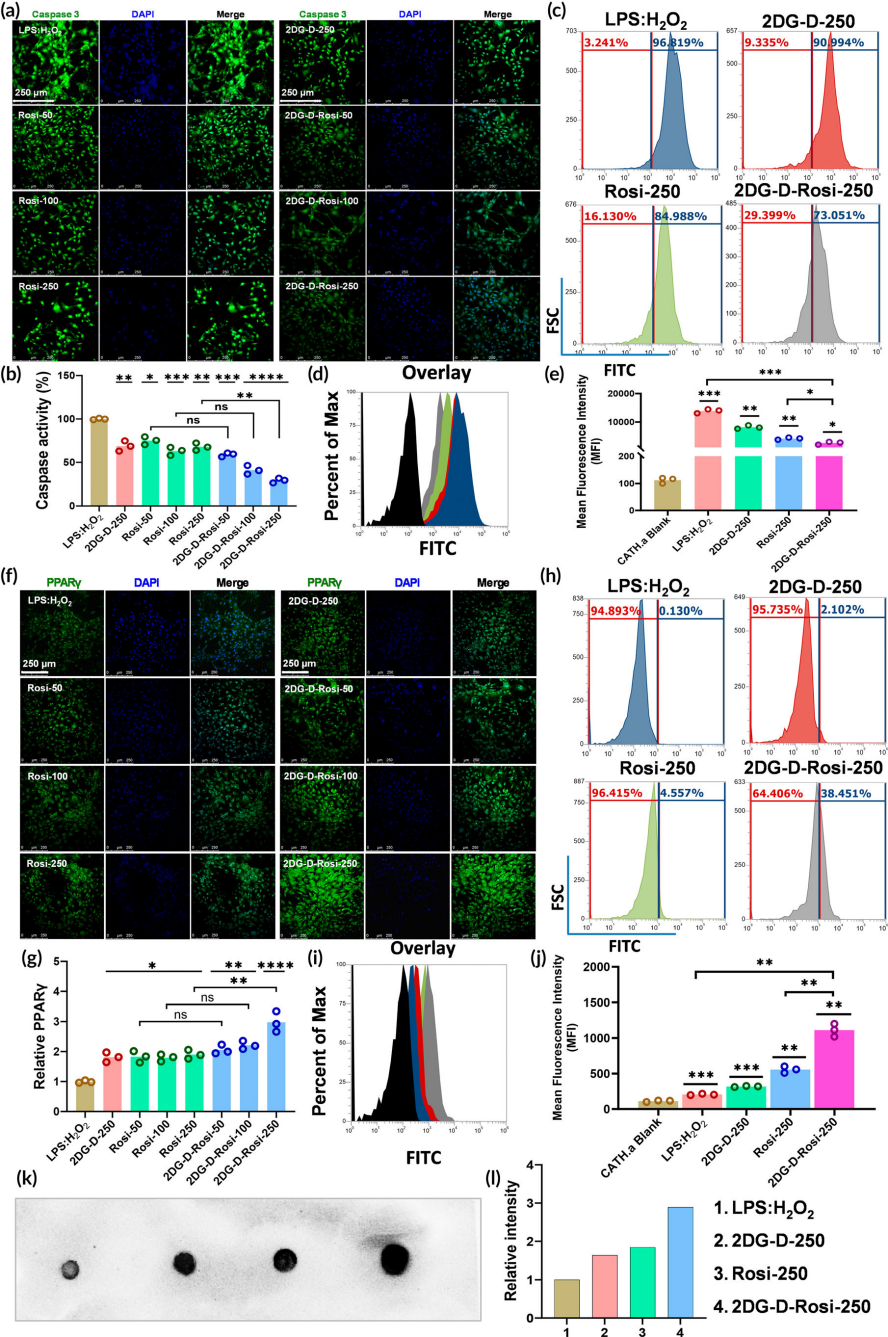
2‐Deoxyglucose‐rosiglitazone (2DG‐D‐Rosi) attenuated caspase activity and upregulated peroxisome proliferator‐activated receptor gamma (PPARγ) expression in CATH.a neurons. (a and b) Confocal microscopy studies: Quantitative and qualitative analysis showing the effect 2DG‐D‐Rosi conjugates on the caspase activity in CATH.a neurons, respectively. (c–e) Flow cytometry studies: (c) Scatter histogram, (d) overlay histogram, and (e) quantitative analysis showing effect of 2DG‐D‐Rosi conjugates on the caspase activity. (f and g) confocal microscopy studies depicting quantitative and qualitative analysis of the effect of 2DG‐D‐Rosi in upregulating the PPARγ expression in cells. (h–j) Flow cytometry studies: (h) scatter histogram, (i) overlay histogram, and (j) quantitative analysis showing effect of 2DG‐D‐Rosi conjugates on the PPARγ upregulation. The effect of 2DG‐D‐Rosi conjugates on the PPARγ expression was further validated using dot blot assay, which shows (k) qualitative and (l) quantitative expression of PPARγ at 250 μg/mL. The scale bars are 250 μm in (a) and (f), the statistical significance was calculated using ordinary one‐way ANOVA, ****p < 0.0001; ***p < 0.001; **p < 0.01; *p < 0.05. DAPI, 4′,6‐diamidino‐2‐phenylindole; H_2_O_2_, hydrogen peroxide; LPS, lipopolysaccharide; ns, nonsignificant.

In the later stages of TBI, apoptosis plays a pivotal role in the pathophysiology of the condition, and inhibiting this process can aid in neuronal recovery.^54^ During TBI, the activation of caspase‐3, the key executioner caspase, contributes to apoptosis and exacerbates neuronal damage. Furthermore, caspase‐3 promotes the proteolysis of DNA repair and cytoskeletal proteins, leading to DNA damage and further apoptosis.^55^, ^56^ To evaluate the effect of 2DG‐D‐Rosi conjugates on alleviating caspase activity, confocal microscopy studies were conducted. Following the induction of TBI‐like conditions, neurons were treated with varying concentrations of 2DG‐D‐Rosi dendrimer conjugates for 24 h, after which confocal microscopy was performed. Quantitative analysis using ImageJ of the confocal images revealed that, compared to the control group (100% caspase activity), caspase activity was reduced in the Rosi‐treated groups (~75%, ~63%, and ~68%) and in the 2DG‐D‐Rosi‐treated groups (~59%, ~42%, and ~ 29%) at concentrations of 50, 100, and 250 μg/mL, respectively. 2DG‐D‐Rosi treatments suppressed caspase activity significantly more than Rosi in a dose‐dependent manner, highlighting its superior efficacy (Figure 6a,b).

The flow cytometry analysis (Figure 6c–e) further confirmed these findings, showing a dramatic increase in caspase activity following LPS:H_2_O_2_ exposure (MFI: ~13,865), compared to baseline levels in untreated cells (MFI: ~112). Treatment with 2DG‐D and Rosi at 250 μg/mL significantly reduced caspase activation (MFI: ~8158 and MFI: ~4149, respectively, *p* < 0.01). Notably, 2DG‐D‐Rosi exhibited the most significant suppression of caspase activity (MFI: ~2583, *p* < 0.001), confirming its enhanced apoptotic inhibition. Compared to free Rosi, which lowered caspase activity by ~70%, 2DG‐D‐Rosi treatment led to a further ~38% reduction (*p* < 0.001), demonstrating its superior caspase‐inhibitory effects. Additionally, the percentage of caspase‐positive cells was significantly lower in the 2DG‐D‐Rosi group compared to all other treatments, further reinforcing its potential to prevent apoptosis. These results emphasize that 2DG‐D‐Rosi more effectively inhibits caspase‐3 activation than free Rosi, suggesting a greater neuroprotective potential in mitigating TBI‐induced apoptotic cell death.

### 3.8 Neuronal stimulation by 2DG‐D‐Rosi upregulates PPARγ expression

PPARγ plays a pivotal role in neuroprotection by regulating neuroinflammation and astrocyte polarization through anti‐apoptotic and anti‐oxidant mechanisms.^15^, ^57^ In this study, we evaluated whether neuronal stimulation with 2DG‐D‐Rosi dendrimer conjugates could modulate PPARγ expression levels. Following treatment with 2DG‐D‐Rosi, a dose‐dependent increase in PPARγ expression was observed, as evident from qualitative confocal micrographs. Quantitative analysis using ImageJ software revealed that compared to the LPS:H₂O₂ treated group (one‐fold), treatment with 2DG‐D at 250 μg/mL resulted in a notable increase in PPARγ expression (1.8‐fold, *p* < 0.05). Furthermore, when comparing the relative levels of PPARγ expression between free Rosi and 2DG‐D‐Rosi at concentrations of 50, 100, and 250 μg/mL, the Rosi‐treated group showed increases of 1.82‐, 1.79‐, and 1.9‐fold, respectively. In contrast, 2DG‐D‐Rosi demonstrated significantly higher PPARγ expression levels of 2.1 (*p* < 0.01), 2.2 (*p* < 0.01), and 3.1‐fold (*p* < 0.01), at 50, 100, and 250 μg/mL (Figures 6f,g and S24). Flow cytometry analysis (Figure 6h–j) further validated these findings, showing that LPS + H_2_O_2_ treatment resulted in a significant reduction in PPARγ expression (MFI: ~206) compared to untreated neurons (MFI: ~112). Treatment with 2DG‐D and Rosi significantly increased PPARγ expression (MFI: ~318 and MFI: ~556, respectively, *p* < 0.01). However, 2DG‐D‐Rosi treatment resulted in a much significant upregulation of PPARγ expression (~1111, *p* < 0.01), demonstrating its superior ability to enhance PPARγ signaling. Compared to free Rosi, 2DG‐D‐Rosi led to nearly a two‐fold greater increase in PPARγ expression (*p* < 0.01), reinforcing the advantage of dendrimer‐based delivery. These results further highlight the enhanced neuroprotective effects of 2DG‐D‐Rosi, which may be linked to its ability to efficiently modulate inflammatory responses and protect against oxidative stress in neurons.

**FIGURE 7:**
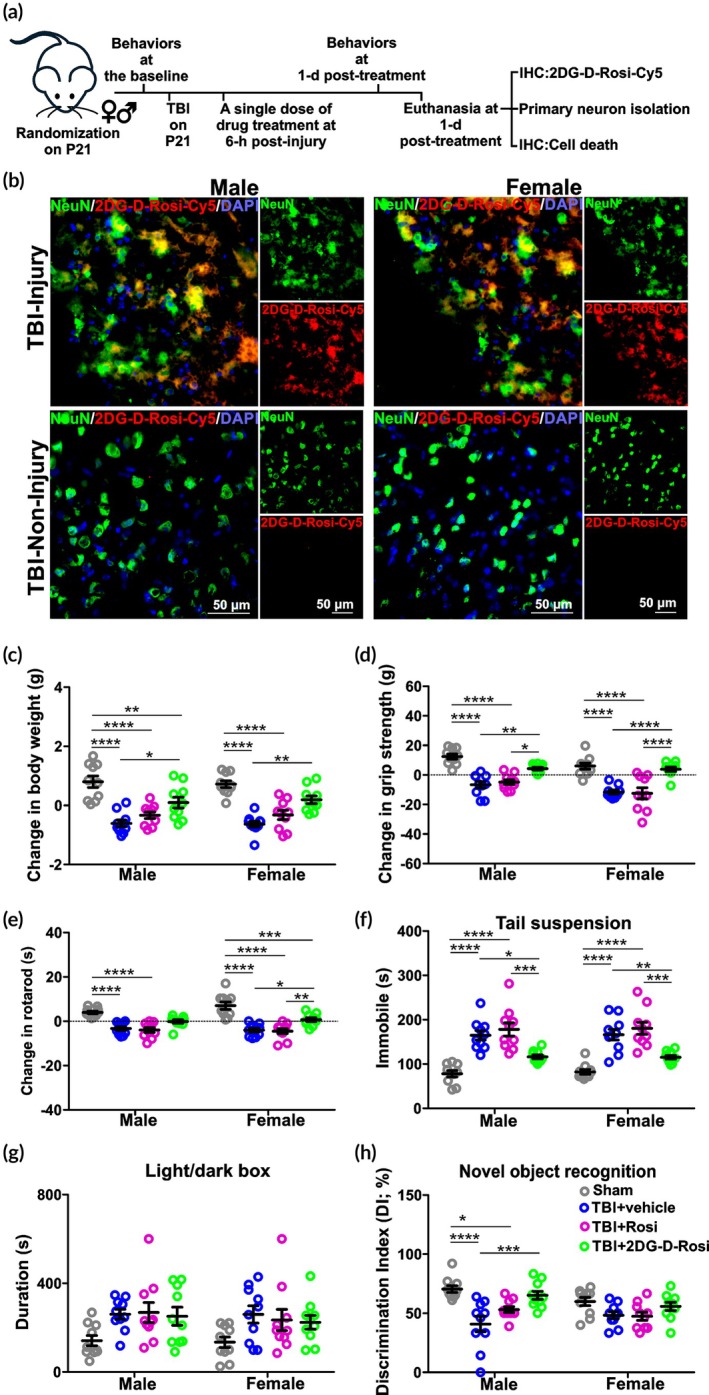
Evaluation 2‐deoxyglucose‐rosiglitazone‐cyanine 5 (2DG‐D‐Rosi‐Cy5) cellular localization and behavioral outcomes in vivo. (a) Schematic illustration of in vivo experimental design. IHC: Immunohistochemistry. (b) Cellular internalization and co‐localization of 2DG‐D‐Rosi‐Cy5: male and female traumatic brain injury (TBI) mice (*n* = 2 per sex) received intraperitoneal administration of 2DG‐D‐Rosi‐Cy5 (50 mg/kg, 100 μL) at 6‐h post‐injury, and euthanized at 24‐h post‐injection. Brain slices containing 2DG‐D‐Rosi‐Cy5 (red) were co‐stained with NeuN (a neuronal marker; green) and 4′,6‐diamidino‐2‐phenylindole (DAPI) (blue). The upper panels represent the co‐localization of 2DG‐D‐Rosi and neurons at the site of injury. The lower panels represent the co‐localization of 2DG‐D‐Rosi and neurons at the non‐injured brain regions. Scale bars: 50 μm. (c–h) Evaluation of behavioral outcomes: Male and female mice littermates from the same litter were randomly divided into four groups (*n* = 20 per group, 10 M/10 F): sham, TBI + vehicle, TBI + Rosi, and TBI + 2DG‐D‐Rosi. Mice in the TBI groups received free Rosi (2 mg/kg, 100 μL), 2DG‐D‐Rosi (containing 2 mg/kg rosiglitazone, 100 μL) or vehicle (100 μL) at 6 h post‐injury. The sham group did not receive any treatment. Behavioral testing was performed at 1 days post‐treatment. Data were presented as mean ± SEM. Treatment groups were presented as sham (gray circles), TBI + vehicle (blue circles), TBI + Rosi (magenta circles), and TBI + 2DG‐D‐Rosi (green circles). (c) The changes in the body weight at 24 h post‐treatment. (d) The grip strength significantly decreased in both male and female TBI + vehicle and TBI + Rosi groups, compared with the male and female sham and TBI + 2DG‐D‐Rosi groups. (e) The change in the latency to the first fall in the Rotarod test. (f) The immobile time significantly increased in both male and female TBI + vehicle and TBI + Rosi groups, compared with the male and female sham and TBI + 2DG‐D‐Rosi groups. (g) The time spent in the light chamber during the light/dark box test. (h) The time spent with the novel object during the novel object recognition test. *p < 0.05; **p < 0.01; ***p < 0.001; ****p < 0.0001.

To further validate the effect of 2DG‐D‐Rosi treatment on neuronal PPARγ expression, we performed dot blot analyses using isolated neuronal proteins. As shown in Figure 7k,l, neurons treated with 2DG‐D‐Rosi exhibited a substantial increase in PPARγ protein levels (~2.9‐fold) compared to control treatments LPS:H_2_O_2_(~1‐fold), 2DG‐D (~1.6‐fold), and Rosi alone (~1.8‐fold). Quantitative evaluation using ImageJ‐based integrated density measurements confirmed these observations, demonstrating that 2DG‐D‐Rosi robustly enhances neuronal PPARγ expression. Thus, our integrated experimental evidence suggests that 2DG‐D‐Rosi is a potential modulator of neuronal PPARγ expression.

### 3.9 2DG‐D‐Rosi‐Cy5 co‐localizes with neurons at the site of injury from systemic administration in a pediatric mouse model of TBI

We have previously demonstrated that systemically administered Cy5 labeled 2DG‐D accumulates in neurons at the site of brain injury. To evaluate if conjugation of Rosi on 2DG‐D maintains this targeting capability, the cellular co‐localization of Cy5 labeled 2DG‐D‐Rosi in the brain of juvenile TBI mice was assessed. We used an impact acceleration TBI model,^26^, ^27^, ^29^ which replicates the pathophysiology that is commonly observed in humans caused by falls.^26^, ^58^ Studies have shown that falls are the leading cause of TBI in children,^59^ thus this model is suitable for secondary insult modeling with the adaptability for mild/moderate injury through alteration of height and/or weight. The impact acceleration TBI model used in this study causes mild to moderate injury. During our initial model characterization, we collected brain samples at different points post‐injury, for example, <5 min (immediately after TBI), 4–6 h, 1 day, 3 days, 7 days, and 3 months. The impact acceleration can cause both focal and diffused injuries, but in general, there were no skull fractures, and the focal lesions may not be apparent immediately (<5 min) after the injury (Figure S25A). However, the primary injury can induce a variety of secondary injuries, including neuroinflammation, ferroptosis, and oxidative stress, which can lead to a progressive cell death/tissue loss and cause both short‐term and long‐term functional impairments.^26^, ^27^, ^28^, ^29^ This provides a basis for using this model to investigate the underlying mechanisms of both short‐term and long‐term disease progression and evaluate the efficacy of drug treatments. Fluorescently labeled 2DG‐D‐Rosi‐Cy5 conjugates were administered at 6 h post‐injury, and animals were euthanized at 24 h post‐injection (Figure 7a). We found that 2DG‐D‐Rosi‐Cy5 was co‐localized with neurons in the injured brain regions, whereas 2DG‐D‐Rosi‐Cy5 uptake in the non‐injured brain regions of the TBI animals was barely detectable (Figure 7b). The accumulation of 2DG‐D‐Rosi in other cell types, such as microglia, astrocytes, and endothelial cells in the injured brain regions was barely detectable (Figure S25C).

### 3.10 2DG‐D‐Rosi improved behavioral outcomes

To evaluate the efficacy of Rosi and 2DG‐D‐Rosi on body weight, we compared the change in body weight before TBI (baseline) and at 1 day post‐treatment. Upon two‐way ANOVA analysis (sex [male, female], treatment [sham, TBI + vehicle, TBI + Rosi, TBI + 2DG‐D‐Rosi]), there were significant differences in the (body weight)_change_ based on treatment (*F*_(3,72)_ = 35.85, *p* < 0.0001). In males, the body weight significantly decreased in TBI + vehicle (*p* < 0.0001), TBI + Rosi (*p* < 0.0001) and TBI + 2DG‐D‐Rosi (*p* < 0.01) groups, compared with the sham group. Moreover, body weight significantly decreased in the TBI + vehicle group, compared with the TBI + 2DG‐D‐Rosi group (*p* < 0.05). In females, body weight significantly decreased in TBI + vehicle (*p* < 0.0001) and TBI + Rosi (*p* < 0.0001) groups, compared with the sham group. Moreover, body weight significantly decreased in the TBI + vehicle group, compared with the TBI + 2DG‐D‐Rosi group (*p* < 0.01) (Figure 7c).

To evaluate the muscle strength and sensorimotor coordination, we compared the changes in grip strength and rotarod performance before (baseline) and at 1 day post‐treatment. For the grip strength test, upon two‐way ANOVA analysis, there were significant differences based on treatment (*F*_(3,72)_ = 40.7, *p* < 0.0001) and sex (*F*_(1,72)_ = 11.33, *p* = 0.0012). Specifically, grip strength significantly decreased in both male and female TBI + vehicle and TBI + Rosi groups, compared with the male and female sham and TBI + 2DG‐D‐Rosi groups (Figure 7d). For the Rotarod test, upon two‐way ANOVA analysis, there were significant differences based on treatment (F_(3,72)_ = 38.45, *p* < 0.0001). In males, the latency to the first fall significantly decreased in both TBI + vehicle (*p* < 0.0001) and TBI + Rosi (*p* < 0.0001) groups, compared with the sham group. In females, the latency to the first fall significantly decreased in TBI + vehicle (*p* < 0.0001), TBI + Rosi (*p* < 0.0001) and TBI + 2DG‐D‐Rosi (*p* < 0.001) groups, compared with the sham groups. In addition, the latency to the first fall significantly decreased in the TBI + vehicle (*p* < 0.05) and TBI + Rosi (*p* < 0.01) groups, compared with the TBI + 2DG‐D‐Rosi group (Figure 7e).

Next, tail suspension and light/dark box tests were used to evaluate the anxiety and depression‐like behaviors at 1‐day post‐treatment. For the tail suspension test, upon two‐way ANOVA analysis, there were significant differences based on treatment (*F*_(3,72)_ = 42.62, *p* < 0.0001). Specifically, the duration of immobility significantly increased in both male and female TBI + vehicle and TBI + Rosi groups, compared with the male and female sham and TBI + 2DG‐D‐Rosi groups (Figure 7f). For the light/dark box test, upon two‐way ANOVA analysis, there were significant differences in the time spent in the light compartment based on treatment (*F*_(3,72)_ = 5.09, *p* = 0.0030) (Figure 7g). The novel object recognition test was used to evaluate cognitive function at 1‐day post‐treatments. Upon two‐way ANOVA analysis, there were significant differences in the discrimination index based on treatment (*F*_(3,72)_ = 12.32, *p* < 0.0001). Specifically, the mice in the male TBI + vehicle (*p* < 0.0001) and TBI + Rosi (*p* < 0.05) groups spent significantly less time with the novel object, compared with the male sham group. In addition, the mice in the male TBI + vehicle group (*p* < 0.001) spent significantly less time with the novel object, compared with the male TBI + 2DG‐D‐Rosi (Figure 7h).

These results demonstrated that 2DG‐D‐Rosi showed a better efficacy in improving behavioral outcomes, compared with the free Rosi. For example, the grip strength performance significantly decreased in both male and female vehicle‐treated and Rosi‐treated TBI animals, but not in the 2DG‐D‐Rosi‐treated group. Moreover, 2DG‐D‐Rosi treatment significantly improved grip strength, compared with free Rosi treatment in both males and females. In addition, 2DG‐D‐Rosi treatment significantly decreased immobile time during the tail suspension test, compared with the TBI + saline and TBI + Rosi groups in both males and females. Whereas Rosi‐treated TBI animals did not show significant improvement in any of the behavioral tests, compared with the vehicle‐treated group.

### 3.11 2DG‐D‐Rosi decreased neuroinflammatory responses and cell death

Previous studies have shown that Rosi exerts neuroprotective effects via the suppression of neuronal autophagy and apoptosis, regulates astrocyte polarization, and attenuates neuroinflammation after TBI.^16^, ^49^, ^60^ However, in most of the studies, Rosi was administered immediately (~5 min) after TBI and requires frequent re‐dosing.^16^, ^49^, ^60^ In a focal cerebral ischemia mouse model, Rosi was given at 2 h post‐injury; however, only the dosage higher than 3 mg/kg can provide neuroprotection against brain infarcts.^61^ In the present study, we chose the 6‐h post‐injury as the initial treatment time point, which reflects the clinical treatment time for TBI patients^62^ and provides the basis for assessment of the therapeutic time window of the 2DG‐D‐Rosi platform.

**FIGURE 8:**
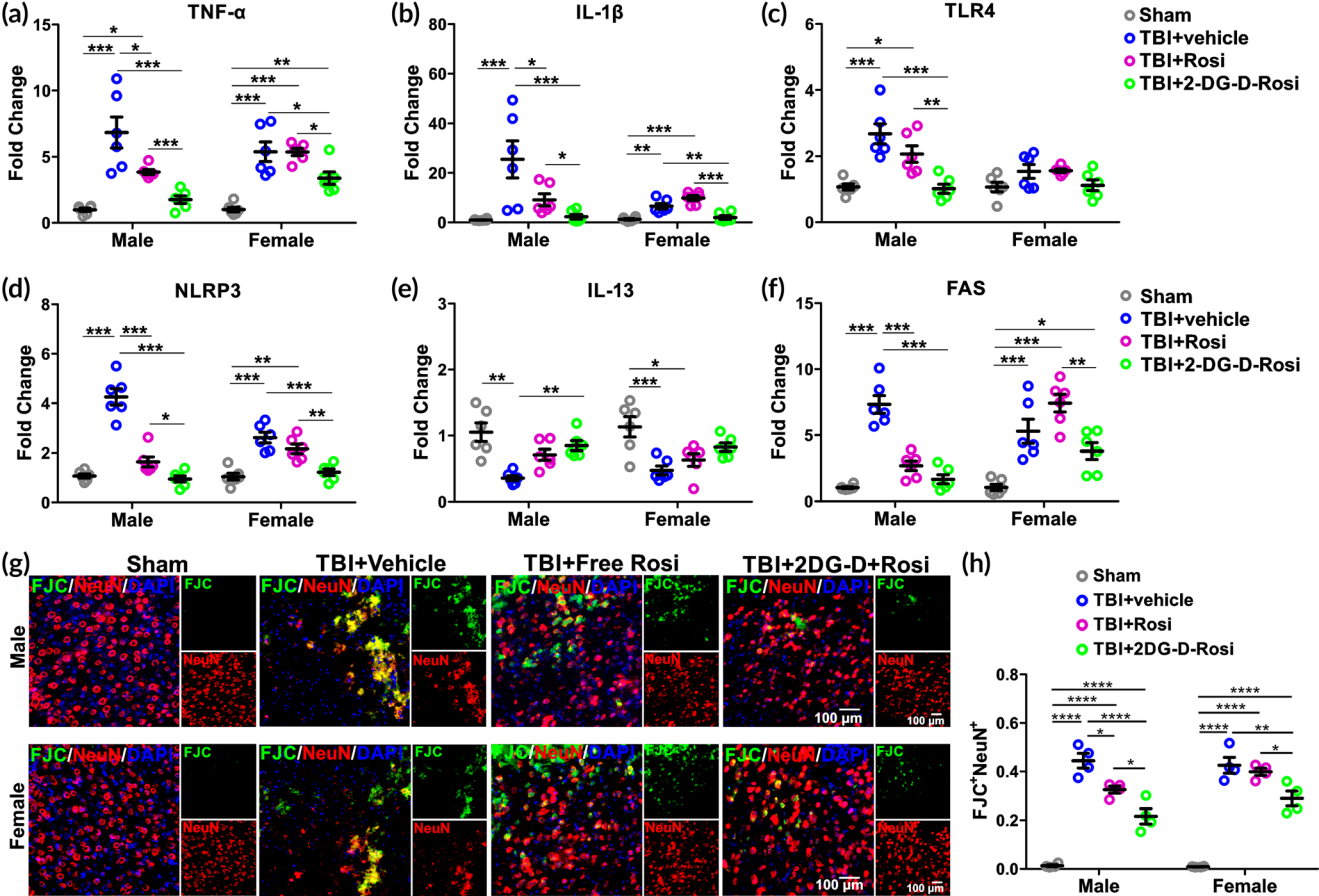
mRNA expressions of pro‐ and anti‐inflammatory markers and cell death markers from sham (*n* = 12, 6 M/6 F), traumatic brain injury (TBI) + vehicle (*n* = 12, 6 M/6 F), TBI + rosiglitazone (Rosi) (*n* = 12, 6 M/6 F), and TBI + 2‐deoxyglucose (2DG‐D)‐Rosi (*n* = 12, 6 M/6 F) groups. Neurons were isolated from the injured brain regions (or the matching area from the sham mice) at 24‐h post‐treatment for gene expression evaluations. Data were presented as mean ± SEM. Treatment groups were presented as sham (gray circles), TBI + vehicle (blue circles), TBI + Rosi (magenta circles), and TBI + 2DG‐D‐Rosi (green circles). (a–d) The expression of pro‐inflammatory makers, tumor necrosis factor‐alpha (TNF‐α) (a), interleukin‐6 beta (IL‐1β) (b), Toll‐like receptor 4 (TLR4) (c), and NLR Family Pyrin Domain Containing 3 (NLRP3) (d). (e) The expression of anti‐inflammatory maker IL‐13. (f) The expression of cell death marker Fas. *p < 0.05; **p < 0.01; ***p < 0.001. (g) The co‐localization of neurodegenerative markers marker Fluoro‐Jade C (FJC) (green) and neuronal marker (NeuN, red) was evaluated in sham (*n* = 8, 4 M/4 F), TBI + vehicle (*n* = 8, 4 M/4 F), TBI + Rosi (*n* = 8, 4 M/4 F), and TBI + 2DG‐D‐Rosi (*n* = 8, 4 M/4 F) groups at 24‐h post‐injury. Images (40×, 5 images/animal) were randomly acquired from the cortex area (mainly primary motor cortex and primary somatosensory cortex) in the injured brain regions (approximately between bregma +1 mm and bregma −0.5 mm) using the Nikon Eclipse TS2R fluorescent microscope (Nikon, NY, USA). The representative images from male treatment groups (upper panels) and female treatment groups (lower panels). 4′,6‐Diamidino‐2‐phenylindole (DAPI) (blue) was used for nucleus stains. Scale bars: 100 μm. (h) The quantification of FJC^+^NeuN^+^ cells among treatment groups. Data were presented as mean ± SEM. Treatment groups were presented as sham (gray circles), TBI + vehicle (blue circles), TBI + Rosi (magenta circles), and TBI + 2DG‐D‐Rosi (green circles). *p < 0.05; **p < 0.01; ****p < 0.0001.

Because 2DG‐D‐Rosi exhibits neuron‐targeting properties, we isolated primary neurons from the injured brain regions (or the matching areas in the sham) to evaluate the efficacy of Rosi and 2DG‐D‐Rosi on neuroinflammatory responses and cell death in neurons. We first compared the mRNA expression of pro‐inflammatory markers (TNF‐α, IL‐1β, TLR4, and NLRP3) at 1 day post‐treatments. Upon two‐way ANOVA analysis (sex [male, female], treatment [sham, TBI + vehicle, TBI + Rosi, TBI + 2DG‐D‐Rosi]), there were significant differences in the TNF‐α expression based on treatment (*F*_(3,40)_ = 33.71, *p* < 0.0001) and the interaction (treatment × sex) (*F*_(3,40)_ = 3.55, *p* = 0.0227). In males, TNF‐α expression significantly increased in TBI + vehicle (*p* < 0.001) and TBI + Rosi (*p* < 0.05) groups, compared with the sham group. TNF‐α expression significantly increased in the TBI + vehicle group, compared with the TBI + Rosi (*p* < 0.05) and TBI + 2DG‐D‐Rosi (*p* < 0.001) groups. Moreover, TNF‐α expression significantly increased in the TBI + Rosi group, compared with the TBI + 2DG‐D‐Rosi group (*p* < 0.001). In females, TNF‐α expression significantly increased in TBI + vehicle (*p* < 0.001), TBI + Rosi (*p* < 0.001) and TBI + 2DG‐D‐Rosi (*p* < 0.01) groups, compared with the sham group. Moreover, TNF‐α expression significantly increased in the TBI + vehicle (*p* < 0.05) and TBI + Rosi (*p* < 0.05) groups, compared with the TBI + 2DG‐D‐Rosi group (Figure 8a). There were significant differences in the IL‐1β expression based on treatment (*F*_(3,40)_ = 11.66, *p* < 0.0001), sex (*F*_(1,40)_=5.01, *p* = 0.0308), and the interaction (treatment × sex) (*F*_(3,40)_ = 5.52, *p* = 0.0029). In males, IL‐1β expression significantly increased in the TBI + vehicle group, compared with sham (*p* < 0.0001), TBI + Rosi (*p* < 0.001), and TBI + 2DG‐D‐Rosi (*p* < 0.0001) groups. Moreover, IL‐1β expression significantly increased in the TBI + Rosi group, compared with the TBI + 2DG‐D‐Rosi group (*p* < 0.05). In females, IL‐1β expression significantly increased in the TBI + vehicle (*p* < 0.01) and TBI + Rosi (*p* < 0.001) groups, compared with the sham group. Moreover, IL‐1β expression significantly increased in the TBI + vehicle (*p* < 0.01) and TBI + Rosi (*p* < 0.001) groups, compared with the TBI + 2DG‐D‐Rosi group (Figure 8b). There were significant differences in the TLR4 expression based on treatment (*F*_(3,40)_ = 16.33, *p* < 0.0001), sex (*F*_(1,40)_ = 8.61, *p* = 0.0055) and the interaction (treatment × sex) (*F*_(3,40)_ = 4.66, *p* = 0.0069). Specifically, TLR4 expression significantly increased in the male TBI + vehicle (*p* < 0.001) and TBI + Rosi (*p* < 0.05) groups, compared with the male sham group. Moreover, TLR4 expression significantly increased in the male TBI + vehicle (*p* < 0.0001) and TBI + Rosi (*p* < 0.001) groups, compared with the TBI + 2DG‐D‐Rosi group (Figure 8c). There were significant differences in the NLRP3 expression based on treatment (*F*_(3,40)_ = 66.63, *p* < 0.0001) and the interaction (treatment × sex) (*F*_(3,40)_ = 12.77, *p* < 0.0001). In males, NLRP3 expression significantly increased in the TBI + vehicle group, compared with the male sham (*p* < 0.0001), TBI + Rosi (*p* < 0.0001), and TBI + 2DG‐D‐Rosi (*p* < 0.0001) groups. Moreover, NLRP3 expression significantly increased in the TBI + Rosi (*p* < 0.05) group, compared with the TBI + 2DG‐D‐Rosi group. In females, NLRP3 expression significantly increased in the TBI + vehicle (*p* < 0.0001) and TBI + Rosi (*p* < 0.001) groups, compared with the sham group. Moreover, NLRP3 expression significantly increased in the TBI + vehicle (*p* < 0.001) and TBI + Rosi (*p* < 0.01) groups, compared with the TBI + 2DG‐D‐Rosi group (Figure 8d).

Next, we compared the mRNA expression of the anti‐inflammatory marker IL‐13 at 1 day post‐treatments. Upon two‐way ANOVA analysis, there were significant differences in the IL‐13 expression based on treatment (*F*_(3,40)_ = 17.19, *p* < 0.0001). In males, IL‐13 expression significantly decreased in the TBI + vehicle group, compared with the sham (*p* < 0.01) and TBI + 2DG‐D‐Rosi (*p* < 0.01) groups. In females, IL‐13 expression significantly decreased in the TBI + vehicle (*p* < 0.001) and TBI + Rosi (*p* < 0.01) groups, compared with the sham group (Figure 8e).

We further compared the mRNA expression of cell death marker Fas at 1‐day post‐treatments. Upon two‐way ANOVA analysis, there were significant differences in the Fas expression based on treatment (*F*_(3,40)_ = 36.24, *p* < 0.0001), sex (*F*_(1,40)_ = 9.56, *p* = 0.0036), and the interaction (treatment × sex) (*F*_(3,40)_ = 13.71, *p* < 0.0001). In males, Fas expression significantly increased in the TBI + vehicle group, compared with the sham (*p* < 0.0001), TBI + Rosi (*p* < 0.0001), and TBI + 2DG‐D‐Rosi (*p* < 0.0001) groups. In females, Fas expression significantly increased in the TBI + vehicle (*p* < 0.001), TBI + Rosi (*p* < 0.001) and TBI + 2DG‐D‐Rosi (*p* < 0.05) groups, compared with the sham group. Moreover, Fas expression significantly increased in the TBI + Rosi group, compared with the TBI + 2DG‐D‐Rosi (*p* < 0.01) group (Figure 8f).

Because 2DG‐D‐Rosi treatment significantly decreased the cell death markers, next, we used the FJC staining to evaluate the neuroprotective effects of 2DG‐D‐Rosi. Upon two‐way ANOVA analysis, there were significant differences in FJC^+^NeuN^+^ cells based on treatment (*F*_(3,24)_ = 126.91, *p* < 0.0001). In males, FJC^+^NeuN^+^ cells significantly increased in the TBI + vehicle (*p* < 0.0001), TBI + Rosi (*p* < 0.0001) and TBI + 2DG‐D‐Rosi (*p* < 0.0001) groups, compared with the sham group. FJC^+^NeuN^+^ cells significantly increased in the TBI + vehicle, compared with the TBI + Rosi (*p* < 0.05) and TBI + 2DG‐D‐Rosi (*p* < 0.0001) groups. Moreover, FJC^+^NeuN^+^ cells significantly increased in the TBI + Rosi (*p* < 0.05) group, compared with the TBI + 2DG‐D‐Rosi group. In females, FJC^+^NeuN^+^ cells significantly increased in the TBI + vehicle (*p* < 0.0001), TBI + Rosi (*p* < 0.0001), and TBI + 2DG‐D‐Rosi (*p* < 0.0001) groups, compared with the sham group. Moreover, FJC^+^NeuN^+^ cells significantly increased in TBI + vehicle (*p* < 0.01) and TBI + Rosi (*p* < 0.05) groups, compared with the TBI + 2DG‐D‐Rosi group (Figures 8g,h and S25D).

Our in vivo results demonstrate that the 2DG‐D‐Rosi treatment significantly decreased pro‐inflammatory markers, such as TNF‐α, IL‐1β and NLRP3 in both males and females, compared with the free Rosi treatment. Studies have shown that pediatric TBI increases production of pro‐inflammatory cytokines such as TNF‐α and IL‐1β, which can exacerbate lipid peroxidation and ionic imbalance, promote cell necrosis, and accelerate neurodegenerative processes.^32^, ^34^, ^63^ The NLRP3 inflammasome plays an important role in the primary inflammatory response to TBI. NLRP3 activation promotes pro‐inflammatory cytokines, such as IL‐1β and IL‐18, which promote the accumulation of ROS and worsen neuroinflammation and oxidative stress, leading to cell death.^64^ The significant anti‐inflammatory effects of 2DG‐D‐Rosi can be responsible for the decreased cell death marker and cell loss.

Interestingly, there are sex differences in the behavioral outcomes and the expression of inflammatory and cell death markers among treatment groups. Studies have shown that there are sex differences in the pharmacokinetics and efficacy of Rosi.^65^ For example, Rosi can stimulate fat growth and improve insulin sensitivity in female mice, but not in males.^65^ Moreover, mounting evidence indicates that TBI alone can induce sex‐specific neuroinflammatory responses^26^, ^29^, ^32^, ^34^, ^63^, ^66^ and behavioral outcomes, depending on differential cellular responses, sex hormones, and metabolism.^67^, ^68^ Therefore, the sex differences in behaviors and neuroinflammatory responses can be caused by the combined effects of brain injury and drug treatment; however, the underlying mechanisms need to be further investigated. Moreover, studies have shown that females exhibit prolonged and extended BBB disruption after TBI compared to males, leading to increased macromolecule and nanoparticle accumulation.^69^ The differences in nanoparticle delivery and behavior depending on sex highlight the need for personalized approaches to TBI treatment.

## 4 CONCLUSIONS

We aimed to develop an effective neuron‐targeted delivery system for Rosi for targeted treatment of TBI. Despite its potential as a therapeutic agent, Rosi faces significant limitations in TBI treatment, including poor solubility, systemic side effects, and targeted delivery to neurons specifically at the site of brain injury. The 2DG‐D‐Rosi delivery platform effectively overcame the challenges of Rosi's poor solubility and limited brain bioavailability. 2DG‐D‐Rosi demonstrated enhanced neuroprotective effects compared to free Rosi in vitro under TBI‐like conditions. The in vivo results demonstrated that by leveraging the dendrimer's targeting capabilities, 2DG‐D‐Rosi achieved precise delivery to injured neurons, reducing neuroinflammation and neuronal loss while improving functional outcomes in a pediatric TBI model. The 2DG‐D‐Rosi nanotherapy and 2DG‐D nanoplatform offer a promising treatment strategy for TBI, with potential applications in other neurodegenerative conditions requiring targeted drug delivery to neurons.

## AUTHOR CONTRIBUTIONS

**Aqib Iqbal Dar**: Data curation; formal analysis; investigation; methodology; project administration; validation; writing—original draft. **Zhi Zhang**: Data curation; formal analysis; funding acquisition; investigation; project administration; resources; supervision; validation; visualization; writing—original draft. **Shamila Gopalakrishnan**: Data curation; formal analysis; methodology; validation; visualization; writing—original draft. **Rishi Sharma**: Conceptualization; formal analysis; investigation; project administration; supervision; validation; visualization; writing—review and editing. **Anunay James Pulukuri**: Data curation; formal analysis; investigation; validation; writing—original draft. **Anu Rani**: Formal analysis; validation; writing—original draft. **Anubhav Dhull**: Data curation; formal analysis; investigation; validation. **Joan Castaneda Gonzalez**: Data curation; formal analysis; validation. **Tia Atoui**: Data curation; formal analysis; validation. **Yara Mashal:** Data curation; formal analysis; validation. **Zahrah Naseer**: Data curation; formal analysis; validation. **Julia Calmi**: Data curation; formal analysis; validation. **Anjali Sharma**: Conceptualization; formal analysis; funding acquisition; investigation; methodology; project administration; resources; software; supervision; validation; visualization; writing—original draft; writing—review and editing.

## FUNDING INFORMATION

This work was partially supported by the Start‐up funds from Department of Chemistry, Washington State University to Anjali Sharma and the “Start‐up” grant (Department of Natural Sciences, CASL, University of Michigan‐Dearborn) for Zhi Zhang.

## CONFLICT OF INTEREST STATEMENT

AS, RS, ZZ, AID, AD, AR have pending patents on 2DG‐dendrimer drug conjugates presented in this manuscript.

## Supporting information


**Data S1.** Supporting Information.

## Data Availability

The data are presented in this manuscript and in Supporting Information S1. Additional data are available upon request.
