# Supplementary material for: Neuron‐targeted 2‐deoxyglucose‐dendrimer‐rosiglitazone nanotherapy mitigates neuroinflammation and cognitive deficits in pediatric traumatic brain injury
Source: Bioeng Transl Med. 2025 Jul 22;11(2):e70053. doi: 10.1002/btm2.70053 (PMC13093522; doi:10.1002/btm2.70053)
Supplement: Supplementary file 1 — Data S1. Supporting Information. [file BTM2-11-e70053-s001.docx]

**Supporting Information**

**Neuron-Targeted 2-Deoxyglucose-Dendrimer-Rosiglitazone Nanotherapy Mitigates Neuroinflammation and Cognitive Deficits in Pediatric Traumatic Brain Injury**

Aqib Iqbal Dar,^1#^ Zhi Zhang,^2#^ Shamila Gopalakrishnan,^1#^ Rishi Sharma,^1^ Anunay James Pulukuri,^1^ Anu Rani,^1^ Anubhav Dhull,^1^ Joan Castaneda Gonzalez,^1^ Tia Atoui,^2^ Yara Mashal,^2^ Zahrah Naseer,^2^ Julia Calmi,^2^ Anjali Sharma^1^*

*^1^Department of Chemistry, College of Arts and Sciences, Washington State University, 1470 NE College Ave, Pullman, WA, USA 99164.*

*^2^Department of Natural Sciences, College of Arts, Sciences, and Letters, University of Michigan -Dearborn, 4901 Evergreen Rd, Dearborn, MI 48128.*

*Correspondence: Anjali Sharma, Department of Chemistry, Washington State University, Troy Hall, Rm 222, 1470 E. College Avenue, Pullman, WA 99164, USA.

E-mail: [anjali.sharma@wsu.edu](mailto:anjali.sharma@wsu.edu)

^#^These authors contributed equally to this work.

**Contents:**

**Supplementary Figures**

**
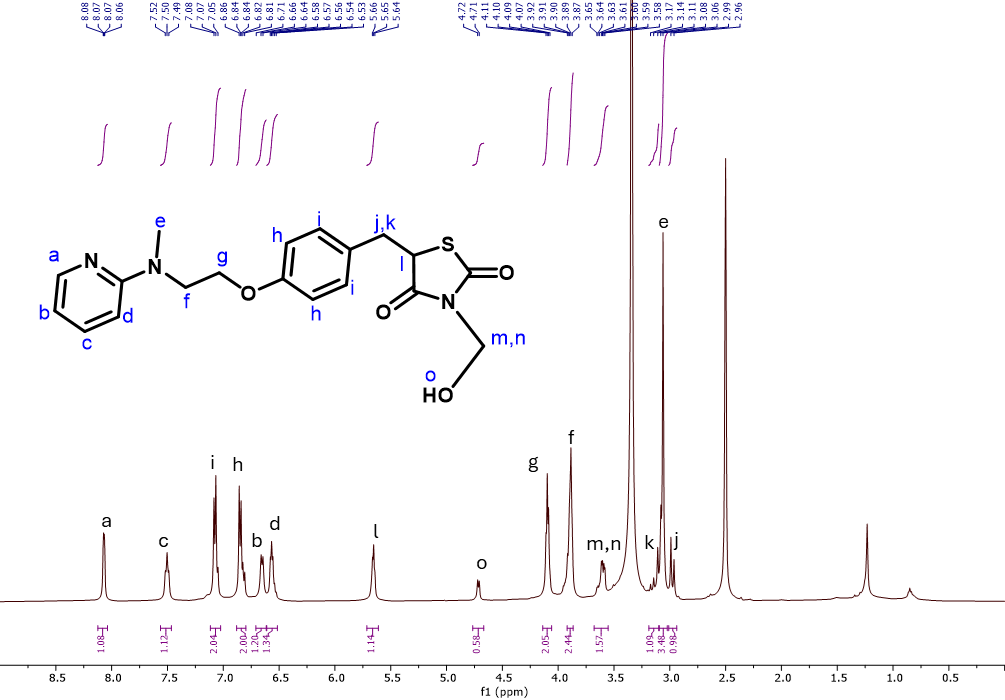
**

**Figure S1:** ^1^H NMR spectrum of compound **2** (DMSO-d_6_, 500 MHz).


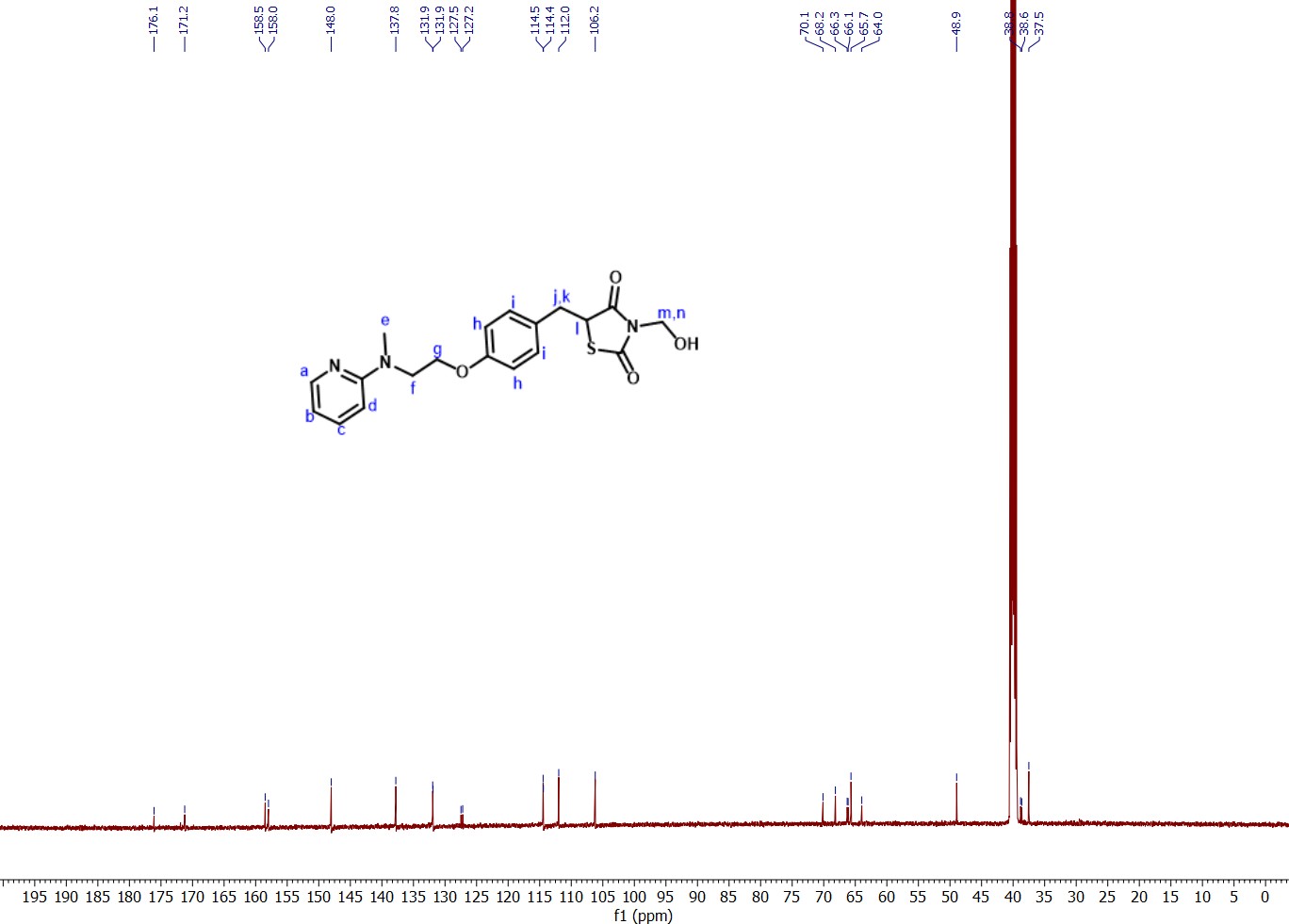


**Figure S2:** ^13^C NMR spectrum of compound **2** (DMSO-d_6_, 125 MHz).


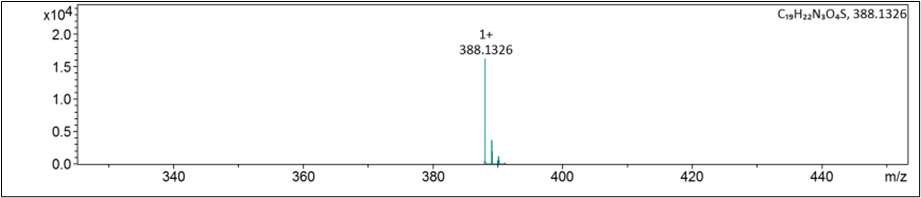


**Figure S3:** Mass spectra of compound **2**.


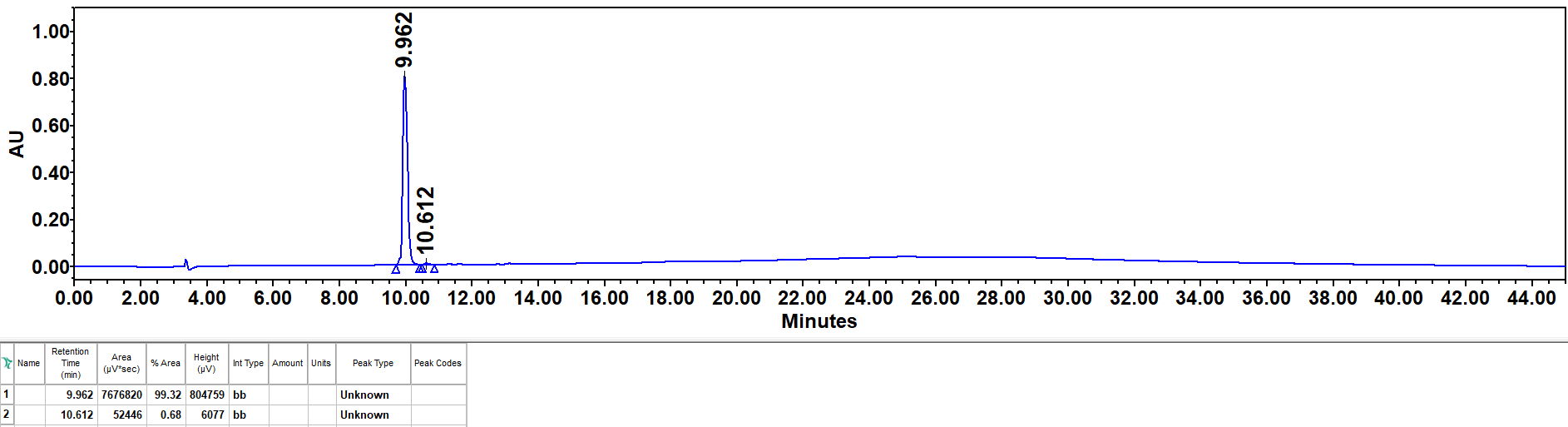


**Figure S4:** HPLC data for compound **2** (purity >99%).

**
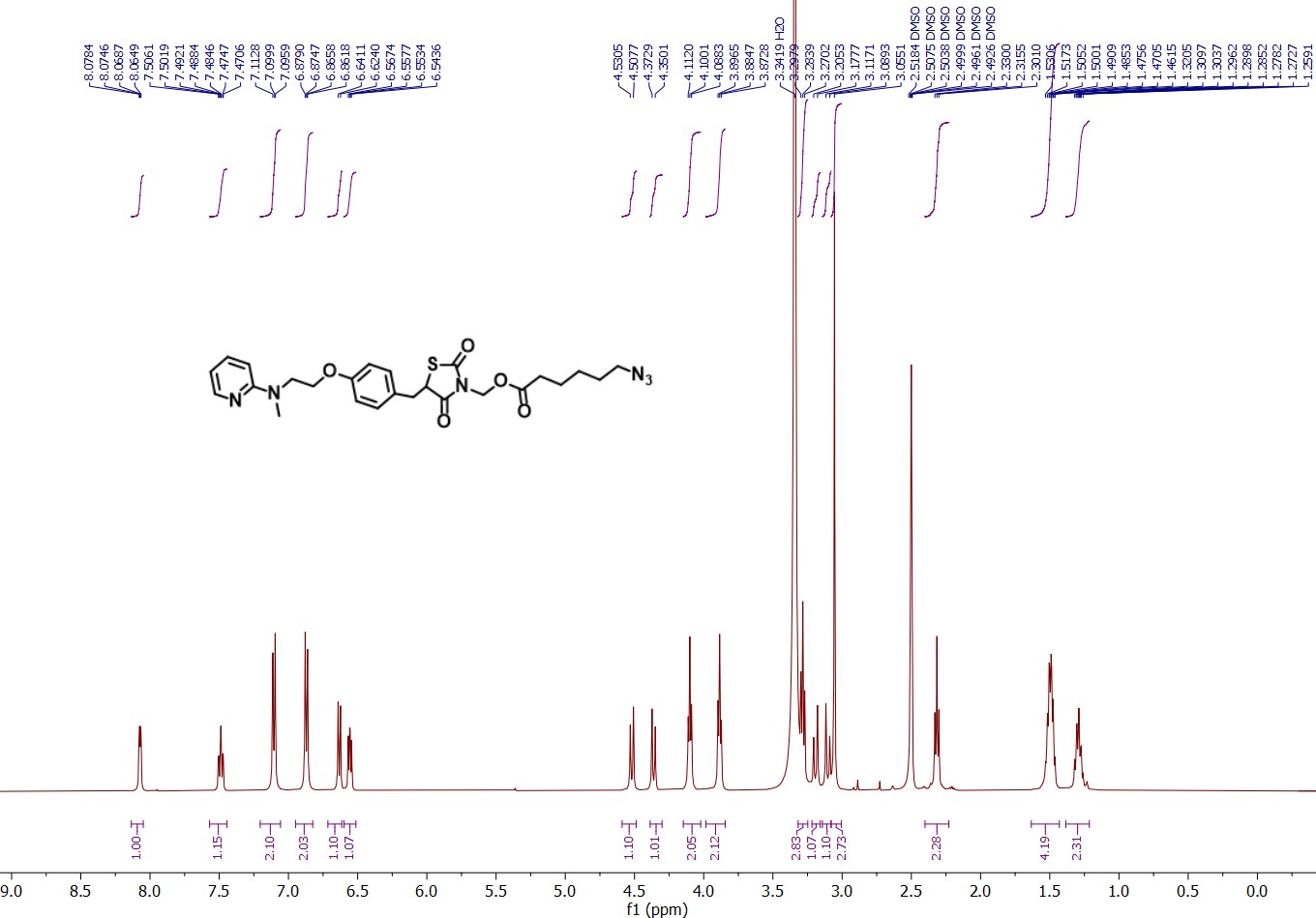
**

**Figure S5:** ^1^H NMR spectrum of compound **4** (DMSO-d_6_, 500 MHz).


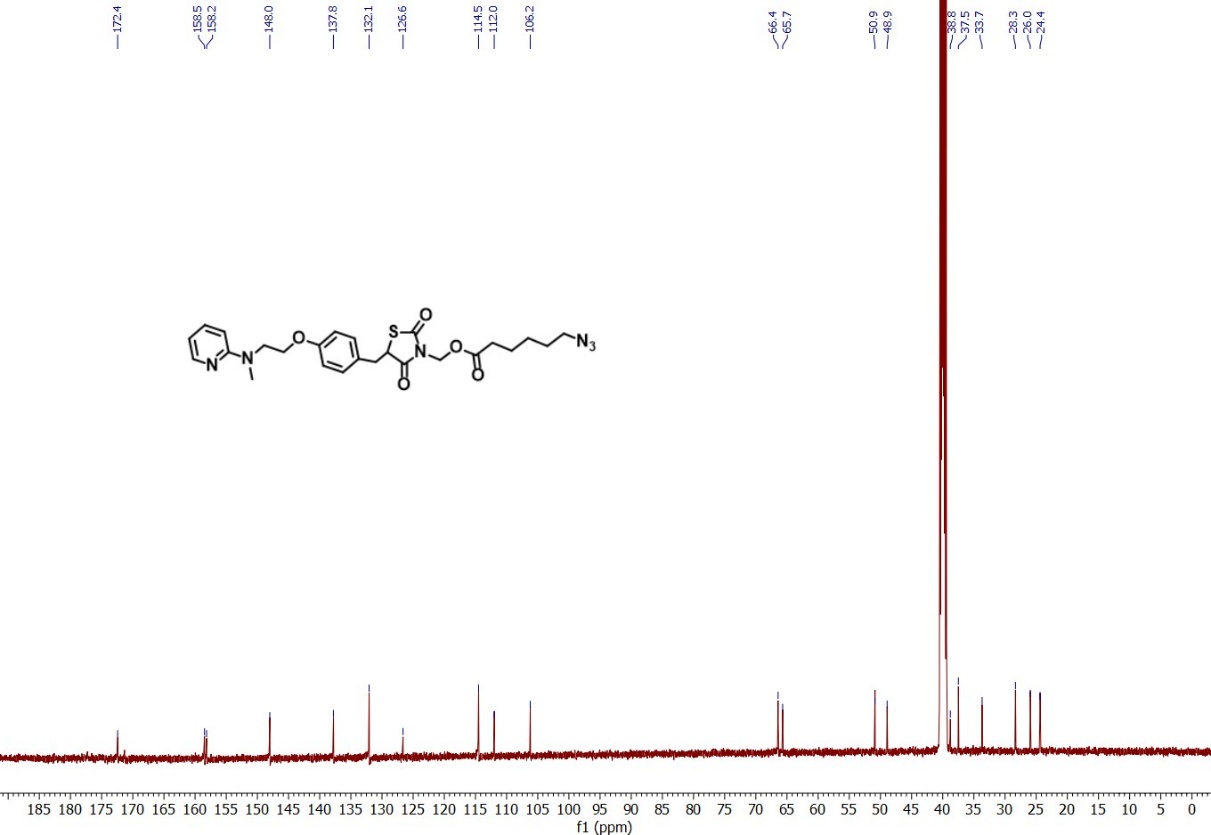


**Figure S6:** ^13^C NMR spectrum of compound **4** (DMSO-d_6_, 125 MHz).

**Figure S7:** Mass spectra (**A**) and HPLC trace (**B**) for compound **4** (purity >99%).


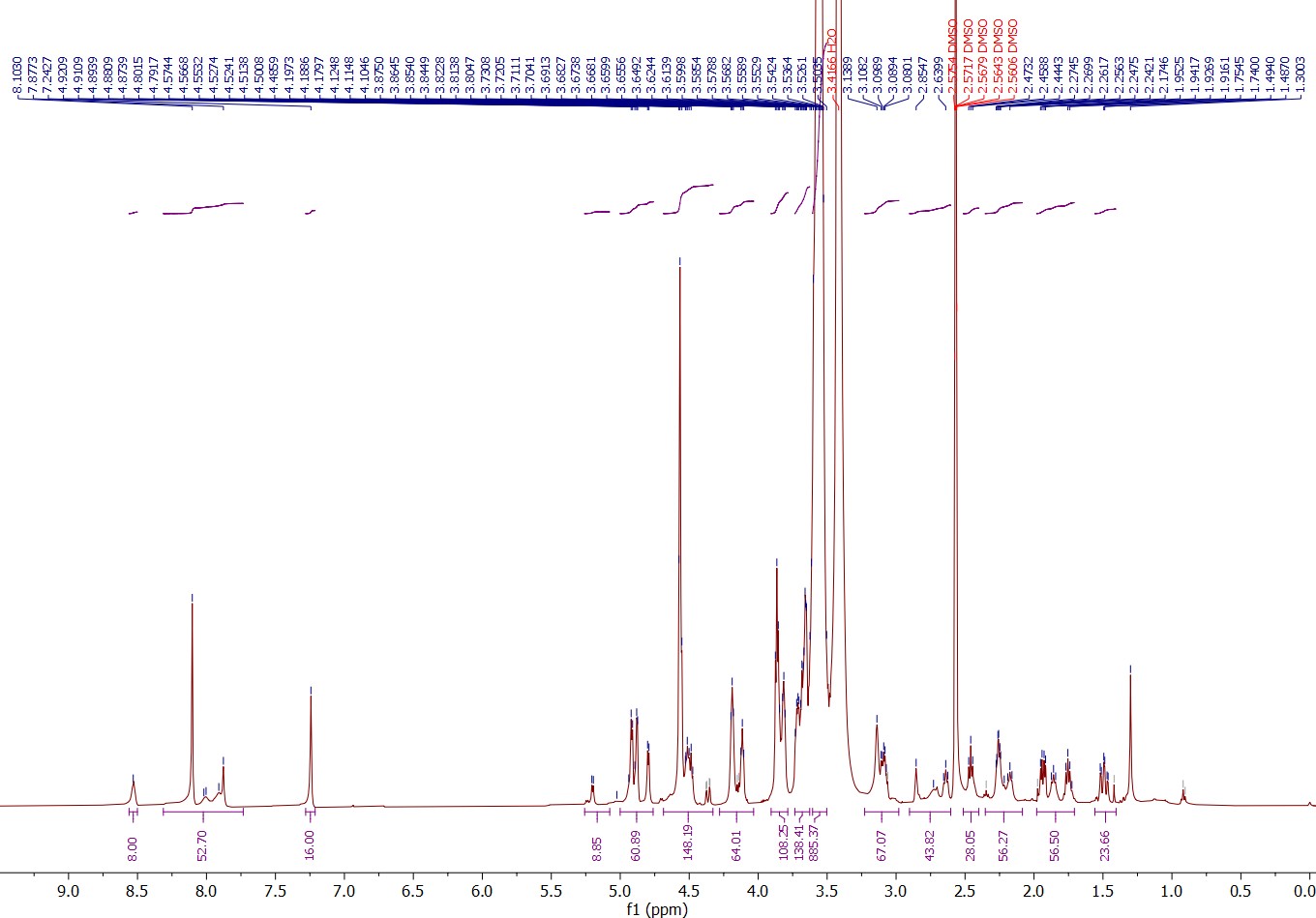


**Figure S8:** ^1^H NMR spectrum of compound **7** (DMSO-d_6_, 500 MHz).


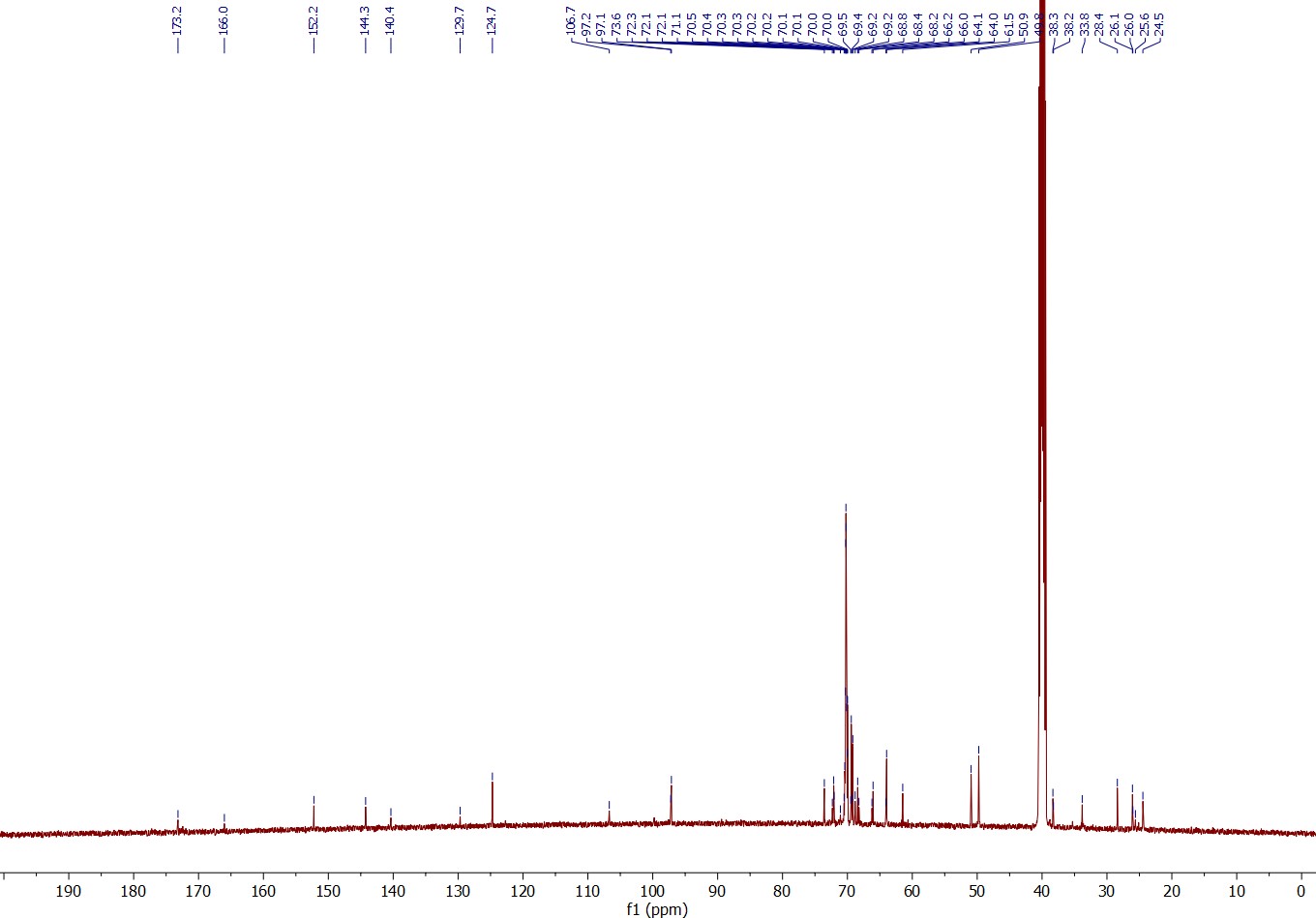


**Figure S9:** ^13^C NMR spectrum of compound **7** (DMSO-d_6_, 125 MHz).


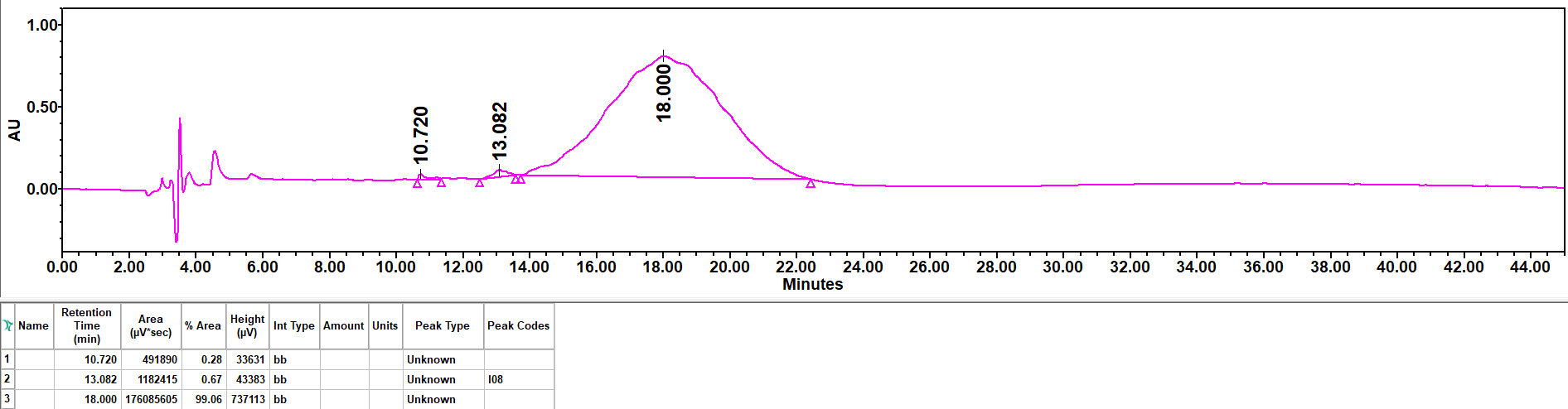


**Figure S10:** HPLC data for compound **7** (Purity 99%).

**Figure S11:** Structure of 2DG-D-Rosi (**8**).


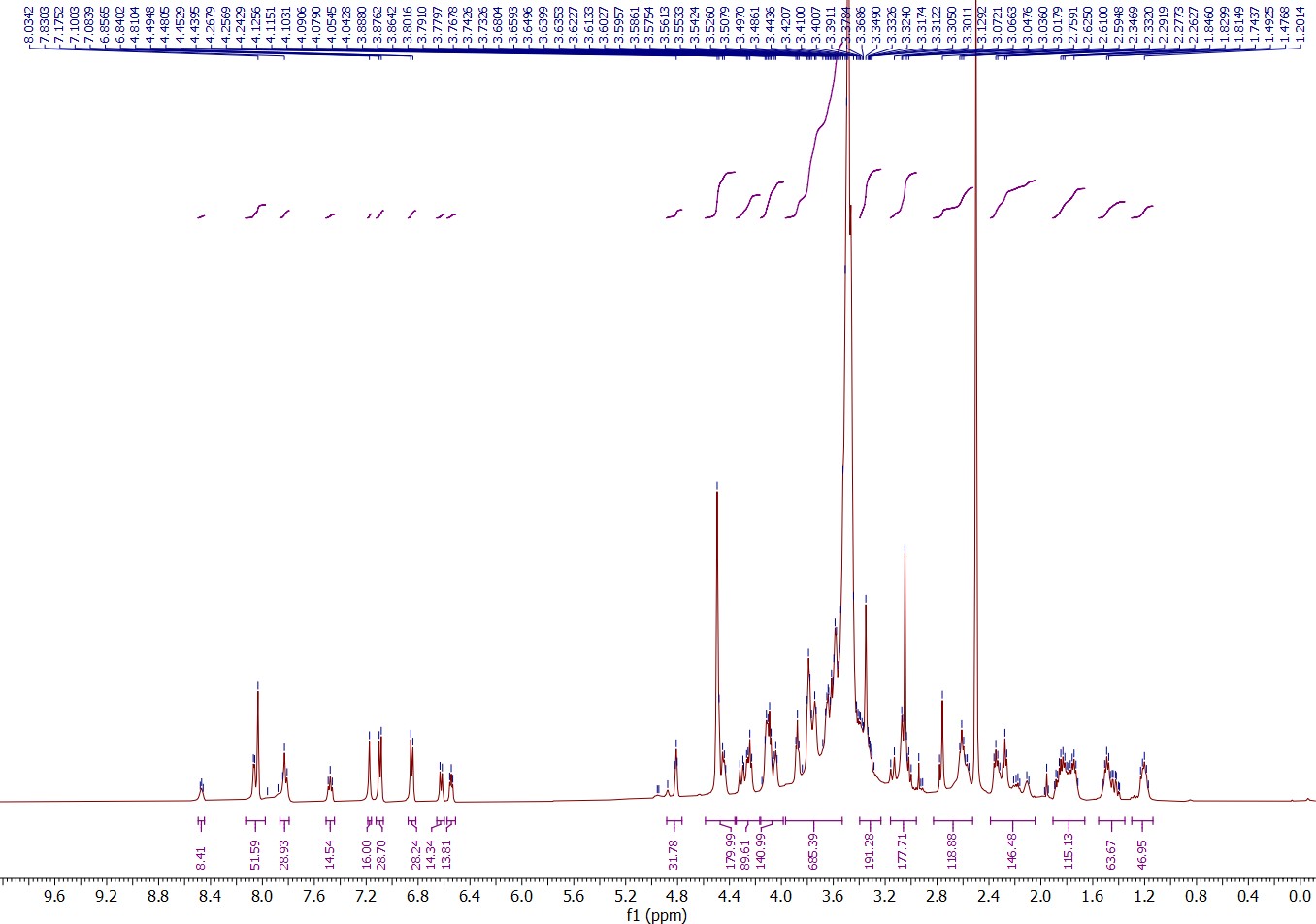
**Figure S12:** ^1^H NMR spectrum of compound **8** (DMSO-d_6_, 500 MHz).


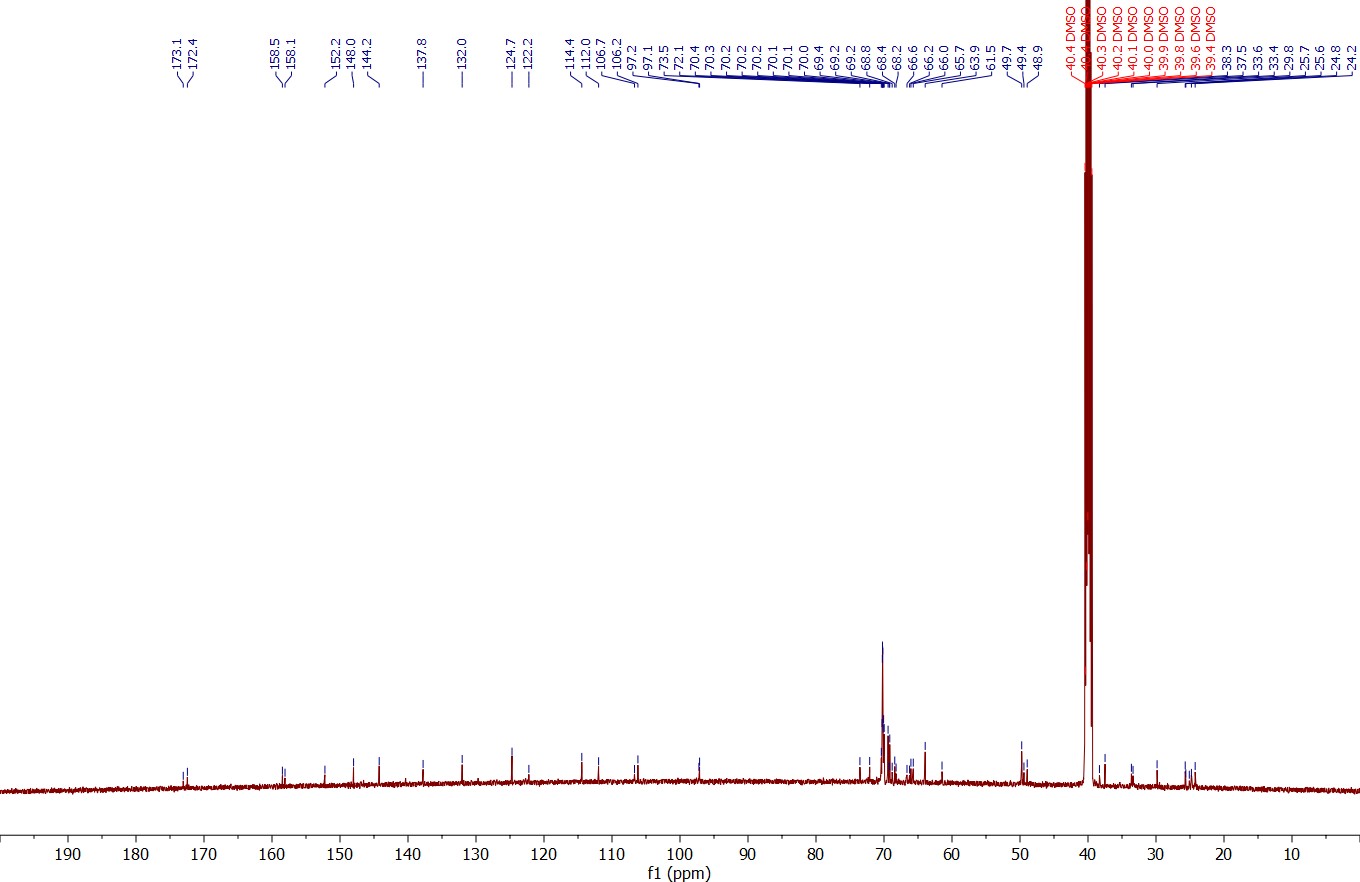


**Figure S13:** ^13^C NMR spectrum of compound **8** (DMSO-d_6_, 125 MHz).


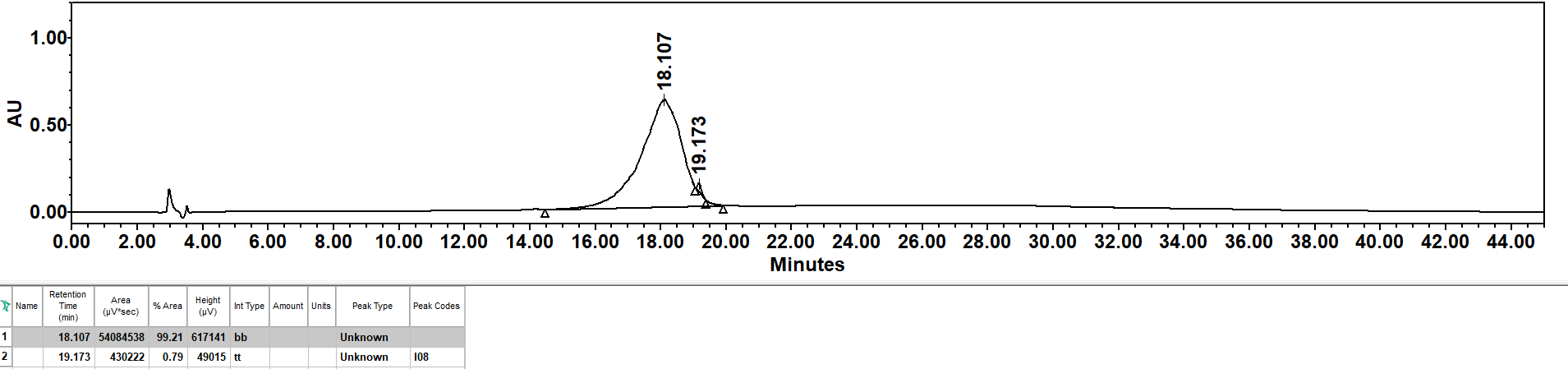
**Figure S14:** HPLC data for compound **8** (Purity 99%).


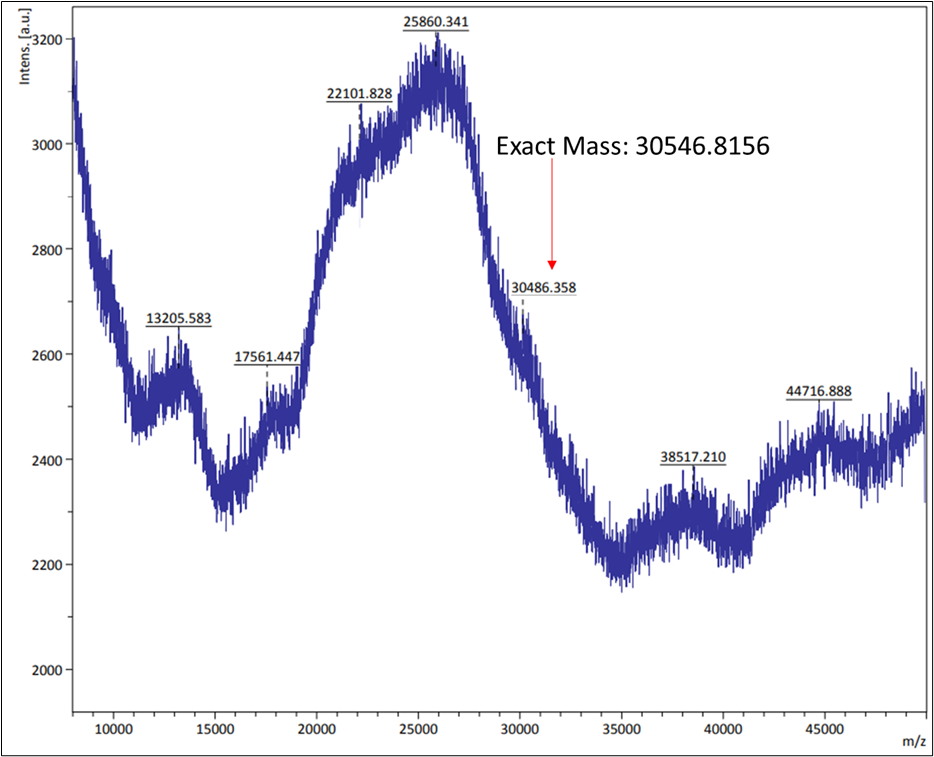


**Figure S15:** Mass spectra of **8**.


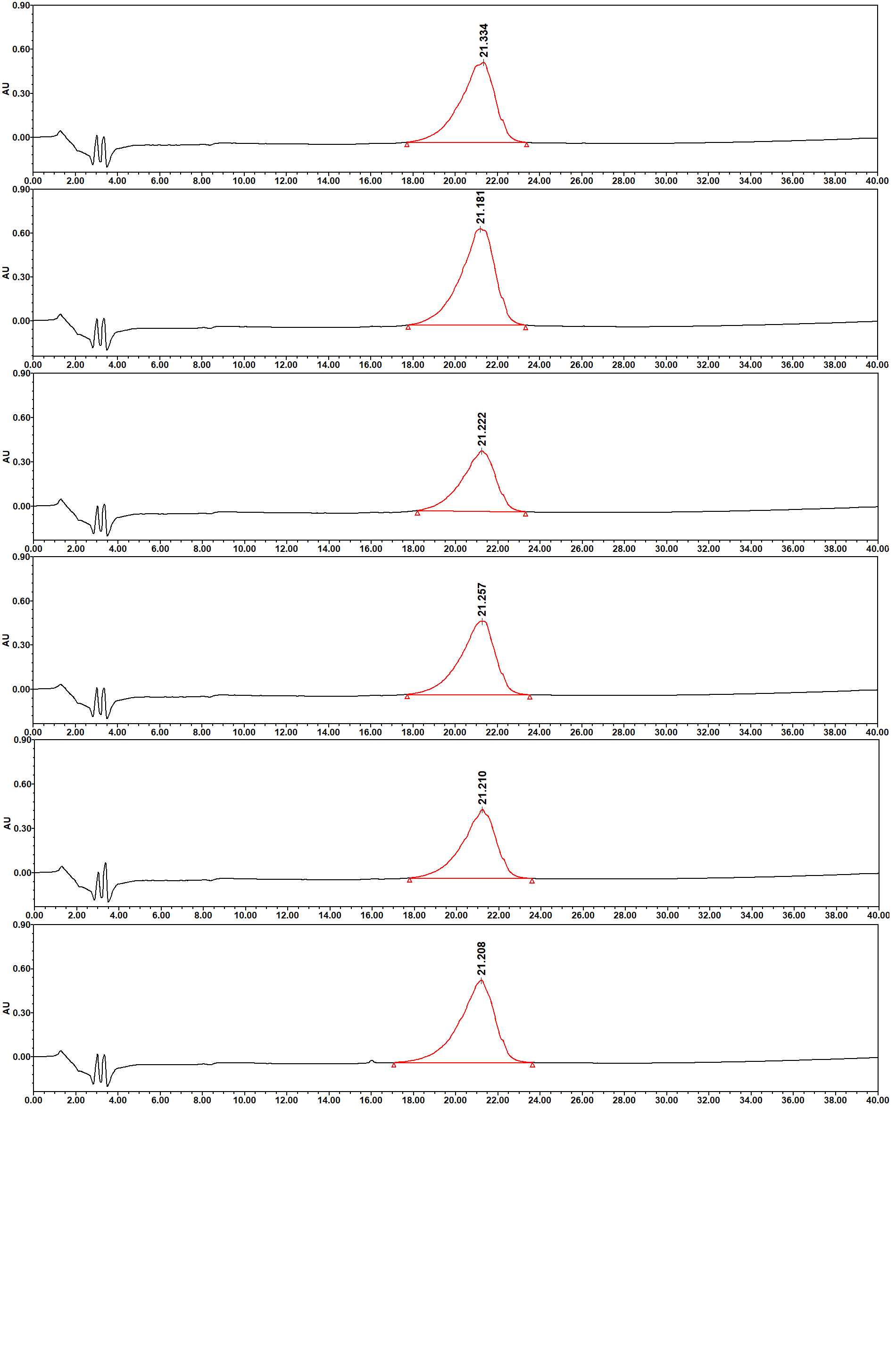


**0 h**

**Purity > 99%**

**Day 1**

**Purity > 99%**

**Day 2**

**Purity > 99%**

**Day 7**

**Purity > 99%**

**Day 15**

**Purity > 99%**

**Day 28**

**Purity > 99%**

**Figure S16:** Formulation stability studies of 2DG-D-Rosi in PBS at 4 °C.


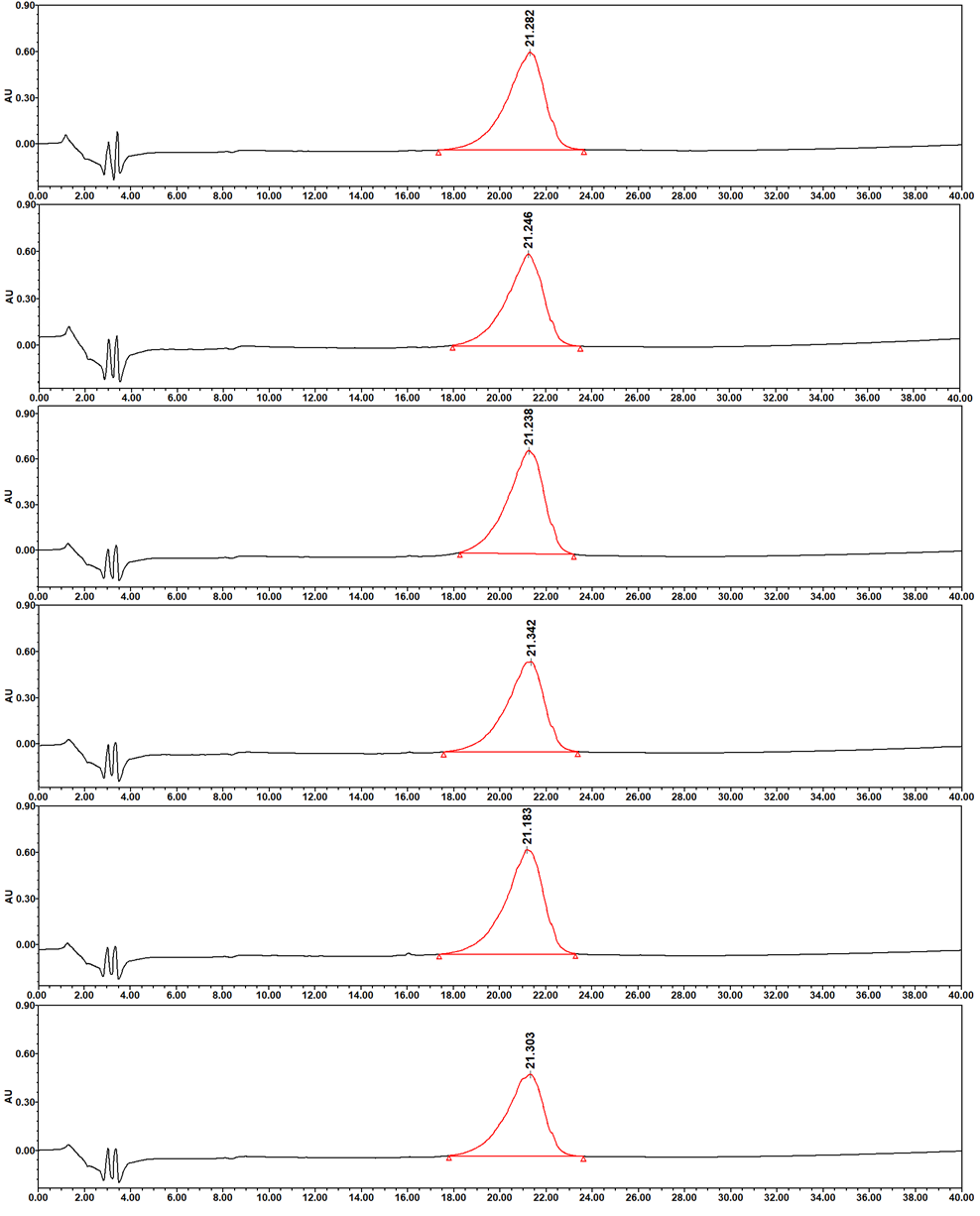


**Day 2**

**Purity > 99%**

**Day 7**

**Purity > 99%**

**Day 15**

**Purity > 99%**

**Day 28**

**Purity > 99%**

**0 h**

**Purity > 99%**

**Day 1**

**Purity > 99%**

**Figure S17:** Formulation stability studies of 2DG-D-Rosi in PBS at 25 °C.


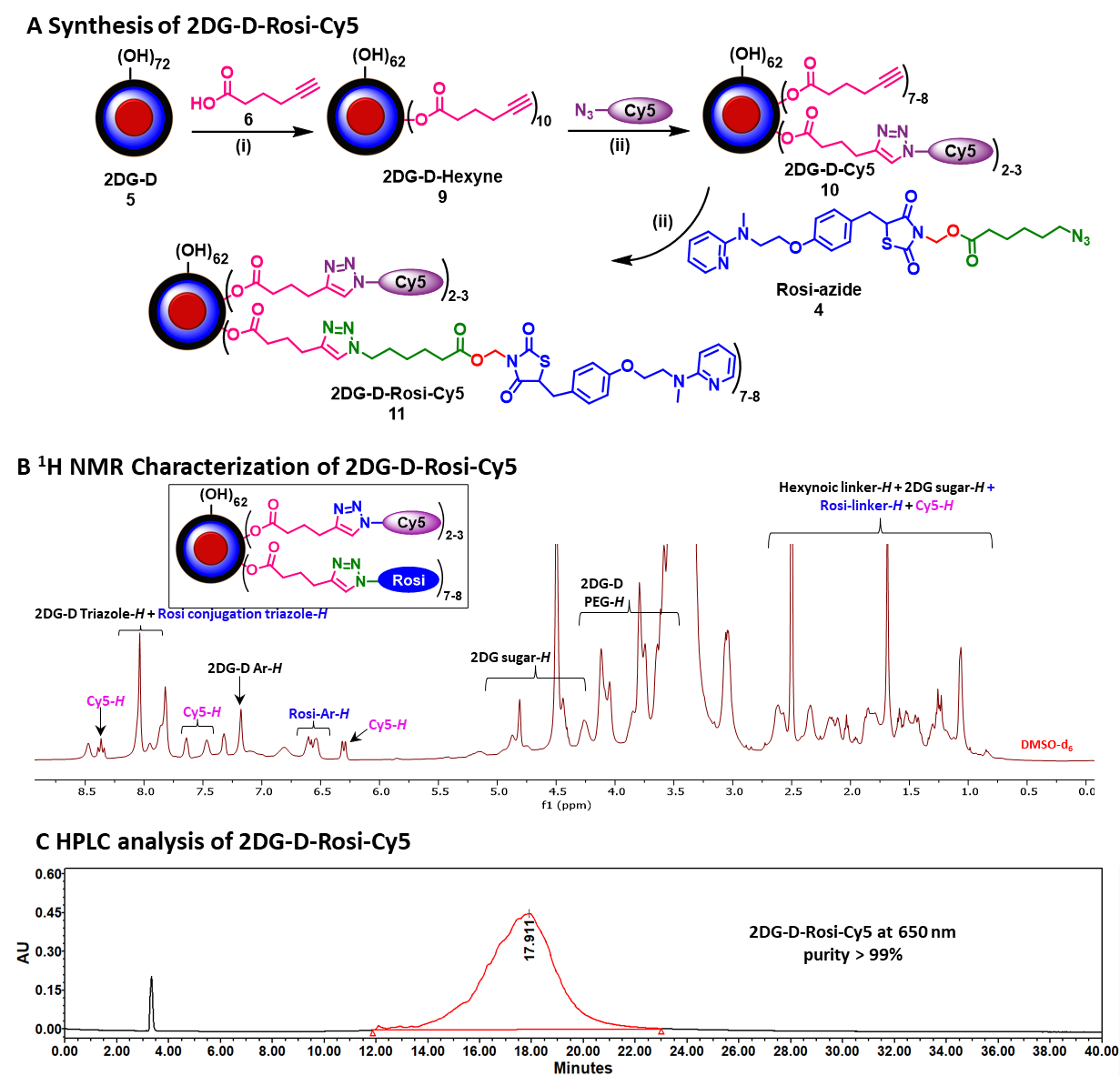


**Figure S18.** **A)** Synthesis route of 2DG-D-Rosi-Cy5. *Reagents and conditions:* (i) EDC.HCl, DMAP, Anhy. DMF, RT, 16 h (ii) CuSO_4_.5H_2_O, Sodium Ascorbate, (DMF: H_2_O = 9:1), RT, 15 h (iii) CuBr, PMDETA, THPTA, Anhy. DMF, RT, 10 h RT. **B)** ^1^H NMR Characterization of 2DG-D-Rosi-Cy5. **C)** HPLC data of 2DG-D-Rosi-Cy5


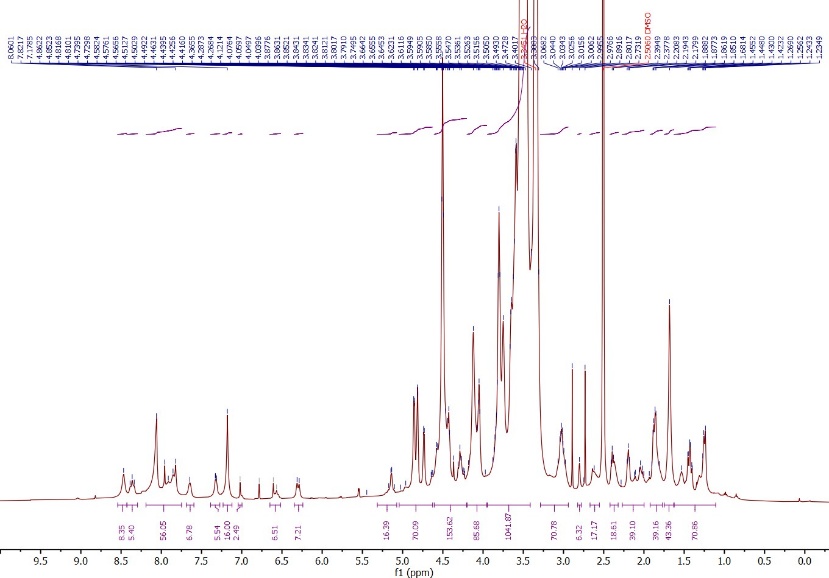


**Figure S19:** ^1^H NMR spectrum of compound **10** (DMSO-d_6_, 500 MHz).

**
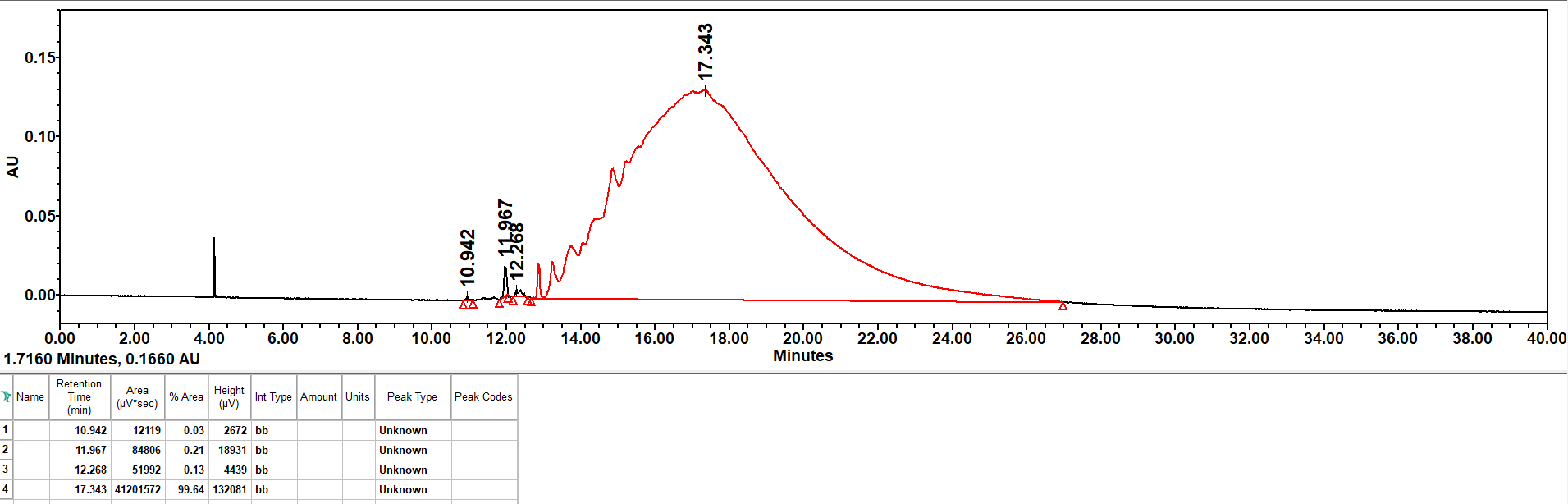
**

**Figure S20:** HPLC data for compound **10** (Purity 99%).

**
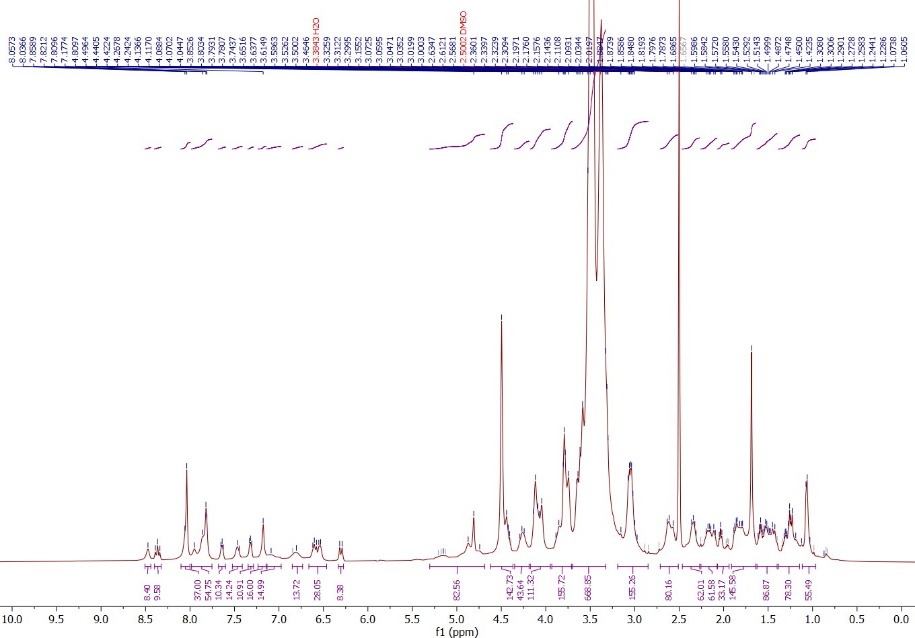
**

**Figure S21:** ^1^H NMR spectrum of compound **11** (DMSO-d_6_, 500 MHz).


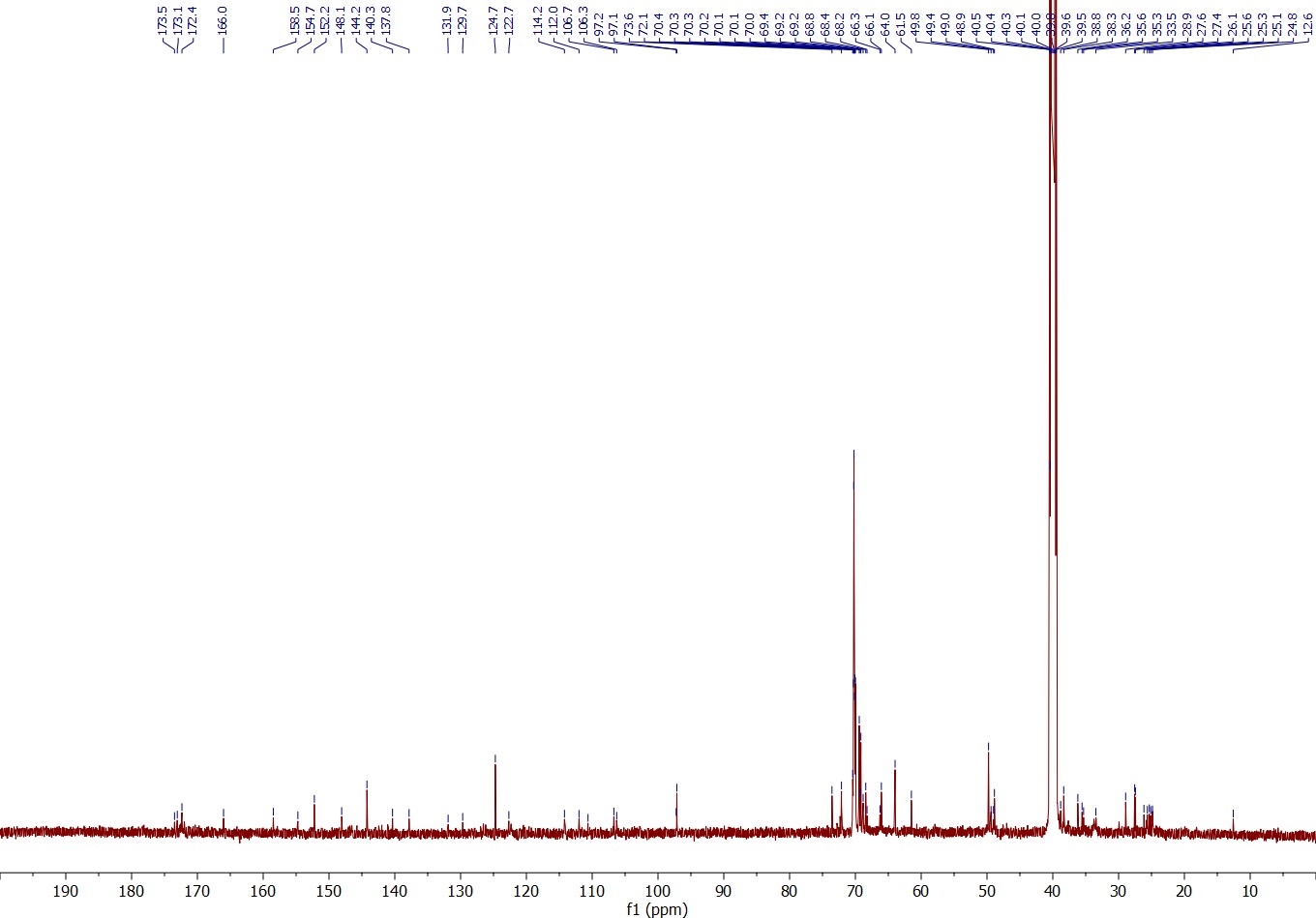
**Figure S22:** ^13^C NMR spectrum of compound **11** (DMSO-d_6_, 125 MHz).

**Figure S23:** Confocal microscopy analysis of untreated control cells depicting the normal polarized MMP as shown by the presence of j-aggregates (red).

**Table S1. Quantitative MMP analysis of 2DG-D conjugate treated CATH.a cells.**

| S.No. | Sample name | Mean Fluorescence Intensity  (Green: J-monomer) | Mean Fluorescence Intensity  (Red: J-aggregate) |
| --- | --- | --- | --- |
| 1 | Control | 2.52 | 201.87 |
| 2 | LPS-H_2_O_2_ | 141 | 2.65 |
| 3 | 2DG-D-250 | 43.14 | 77.1 |
| 4 | Rosi-50 | 89.2 | 8.85 |
| 5 | Rosi-100 | 74.18 | 79.28 |
| 6 | Rosi-250 | 71.68 | 29.83 |
| 7 | 2DG-D-Rosi-50 | 23.63 | 81.72 |
| 8 | 2DG-D-Rosi-100 | 23.29 | 143.02 |
| 9 | 2DG-D-Rosi-250 | 2.35 | 174.1 |

**Figure S24.** Confocal laser microscopy-based immunofluorescence assay showing PPARγ expression (green) in control untreated cells under *in vitro* conditions.


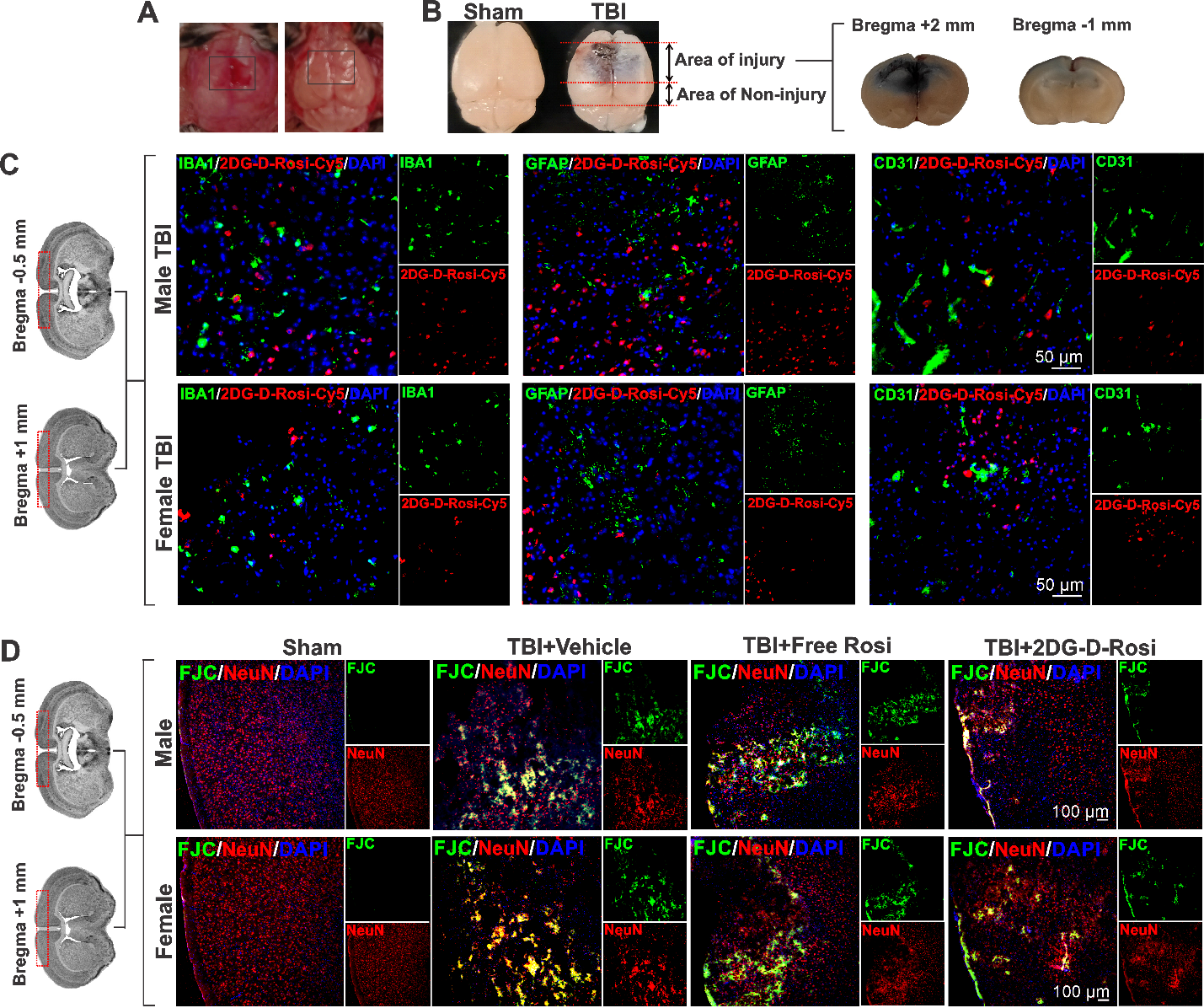


**Figure S25**. The *in vivo* TBI model. **A,** The animal underwent TBI and was euthanized immediately post-TBI (<5 min). The rectangle indicates the location where the falling weight contacted the animal’s head. The left panel shows the animal’s head with the skull. The right panel shows the brain with the skull removed. **B,** The TBI animal (right) underwent TBI, and the sham animal (left) underwent anesthesia without injury. The sham and TBI animals received a single dose (i.p.) of 10 mg/kg of 2% sterile Evans blue (Cat# A16774.18; Thermo Fisher Scientific, MA, USA) at 6-h post-TBI and euthanized at 2 h post-Evans Blue injection. The area of injury was mainly located between the bregma +2 mm and bregma -1 mm. The two brain slices show the normal morphological brain features (without injury) at these coordinates (bregma +2 mm and bregma -1 mm). **C,** Brain slices containing 2DG-D-Rosi-Cy5 (red) were co-stained with IBA1 (a microglia marker; green; the left panels), GFAP (an astrocyte marker; green; the middle panels) or CD31 (an endothelial marker; green; the right panels) and DAPI (blue). Images (40X) were randomly acquired from the cortex area (mainly primary motor cortex and primary somatosensory cortex) in the injured brain regions (approximately between bregma +1 mm and bregma -0.5 mm). The upper panels were from the male TBI animal, and the lower panels were from the female TBI animal. Scale bars: 50 µm. **D,** The co-localization of neurodegenerative marker FJC (green) and neuronal marker (NeuN, red) was evaluated at 24-h post-injury. Images (10X) were randomly acquired from the cortex area (mainly primary motor cortex and primary somatosensory cortex) in the injured brain regions (approximately between bregma +1 mm and bregma -0.5 mm). The representative images from male treatment groups (upper panels) and female treatment groups (lower panels). DAPI (blue) was used for nucleus stains. Scale bars: 100 µm.

**Table S2.** Rotarod dataset
